# GOLM1 exacerbates CD8^+^ T cell suppression in hepatocellular carcinoma by promoting exosomal PD-L1 transport into tumor-associated macrophages

**DOI:** 10.1038/s41392-021-00784-0

**Published:** 2021-11-19

**Authors:** Jinhong Chen, Zhifei Lin, Lu Liu, Rui Zhang, Yan Geng, Minghao Fan, Wenwei Zhu, Ming Lu, Lu Lu, Huliang Jia, Jubo Zhang, Lun-Xiu Qin

**Affiliations:** 1 Department of General Surgery, Huashan Hospital, Cancer Metastasis Institute, Fudan University, 12 Urumqi Road (M), Shanghai, 200040 China; 2 Department of Infectious Diseases, Huashan Hospital, Fudan University, 12 Urumqi Road (M), Shanghai, 200040 China

**Keywords:** Cancer microenvironment, Tumour immunology

## Abstract

The immunosuppressive microenvironment plays an important role in tumor progression and immunotherapy responses. Golgi membrane protein 1 (GOLM1) is correlated to hepatocellular carcinoma (HCC) progression and metastasis. However, little is known about the role of GOLM1 in regulating the immunosuppressive environment and its impact on immunotherapeutic efficacy in HCC. In this study, GOLM1 was positively correlated with infiltrating tumor-associated macrophages (TAMs) expressed high levels of programmed death-ligand 1 (PD-L1) and CD8^+^ T cell suppression in HCC tissues. Both gain- and loss-of-function studies determined a close correlation between GOLM1 and immunosuppression. In the mechanism, GOLM1 promoted COP9 signalosome 5-mediated PD-L1 deubiquitination in HCC cells and increased the transport of PD-L1 into exosomes via suppression of Rab27b expression. Furthermore, co-culture with exosomes derived from HCC cells upregulated the expression of PD-L1 on macrophages. Zoledronic acid in combination with anti-PD-L1 therapy reduced PD-L1^+^ TAMs infiltration and alleviated CD8^+^ T cell suppression, resulting in tumor growth inhibition in the mouse HCC model. Together, our study unveils a mechanism by which GOLM1 induces CD8^+^ T cells suppression through promoting PD-L1 stabilization and transporting PD-L1 into TAMs with exosome dependent. Targeting PD-L1^+^ TAM could be a novel strategy to enhance the efficacy of anti-PD-L1 therapy in HCC.

## Introduction

Hepatocellular carcinoma (HCC) is the fourth most common cause of cancer-related mortality, and hence, is an increasing public concern worldwide^1,2^. Only a small number of HCC patients are eligible for curative treatments, such as surgical resection, liver transplantation, and local ablation, because most patients with HCC have already progressed to advanced stages and metastasis at the time of diagnosis^3,4^. The advent of immune checkpoint blockade (ICB) has revolutionized cancer therapeutics. Several ICB monotherapies, such as programmed death receptor 1 (PD-1) monotherapy and programmed death-ligand 1 (PD-L1) monotherapy, have been approved for the treatment of advanced HCC^5–8^. However, the therapeutic efficacy of ICB therapy is limited by the tumor immune microenvironment, with only 16–20% objective response rates among advanced HCC patients^9^. Thus, analyzing the immune microenvironment of HCC could provide insights into immune evasion mechanisms, which might be helpful to develop more effective immunotherapeutic strategies for HCC.

Intercellular crosstalk in the tumor microenvironment plays a critical role in cancer progression. Classical crosstalk includes direct cell-to-cell contact and secretion of soluble factors, such as growth factors and cytokines. Exosomes are an efficient type of intercellular signal delivery system in the proximal and distant sites and play a key role in cancer progression. These nanovesicles with lipid bilayer membrane are comprised of proteins, lipids, and nucleic acids, derived from multivesicular bodies^10,11^. Exosome-mediated intercellular communication between cancer cells and stromal cells is critical in remolding microenvironment favoring tumor progression^12^.

Upregulation of PD-L1 is an important mechanism implicated in tumor immune escape. PD-L1 is one of the two ligands for PD-1 and is expressed on the surface of cancer cells, dendritic cells (DCs), monocytes, and macrophages^13–15^. The ligation of PD-L1 on cancer cells to PD-1 expressed on T cells induces T cell suppression and blocks further immune rejection, bypassing immune surveillance. The upregulation of PD-L1 shows a correlation with the poor prognosis of HCC^16^. Typically, tumor cell-surface PD-L1 direct interacts with PD-1 on the surface of T cells in the tumor environment. However, some recent studies showed that most tumor cells do not express high levels of PD-L1, while inflammatory cells express higher levels of PD-L1. And in some cancers, PD-L1 expression on myeloid cells, such as macrophages and monocytes, is more effective than tumor cells expressed PD-L1 in suppressing T cell function^17,18^. Moreover, PD-L1 also can be found on the surface of exosomes, and the exosome can deliver PD-L1 into other cell types in the tumor microenvironment^19,20^. Whether exosomal PD-L1 interacts with stromal cells and induces local immunosuppression deserves further study.

Golgi membrane protein 1 (GOLM1) is a type II transmembrane protein originally located in the Golgi apparatus and cycles among membranous compartments, such as sorting endosomes and the plasma membrane. In our previous study, GOLM1 was identified as one of the leading genes that is significantly upregulated in metastatic HCC. It promotes HCC metastasis by facilitating epidermal growth factor receptor (EGFR) endocytosis and recycling in endosome-like structure^21^. In the present study, we further investigated the role of GOLM1 in regulating the immunosuppressive microenvironment of HCC and explored the underlying mechanisms.

## Results

### Overexpression of GOLM1 is associated with immunosuppressive microenvironment in HCC and immune-escape of tumor cells

**Fig. 1:**
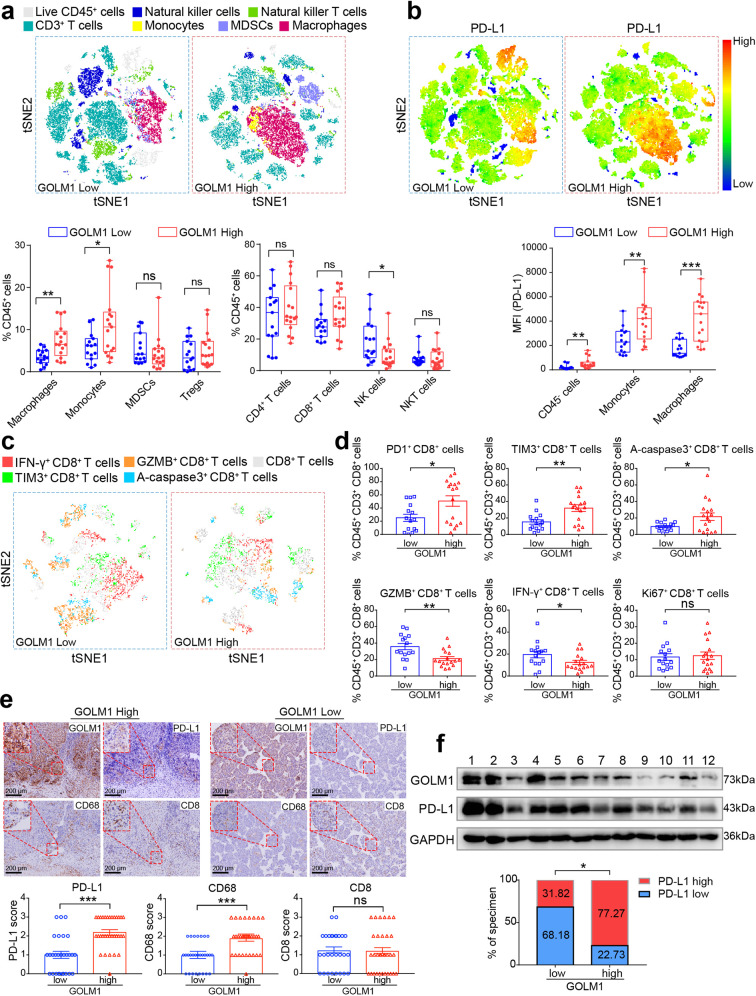
Overexpression of GOLM1 is associated with the immunosuppressive microenvironment in HCC and immune-escape of tumor cells. **a** Differences in the immunophenotypes of infiltrating immune cells detected by flow cytometry in HCC tissues with high or low GOLM1 expression. t-Distributed Stochastic Neighbor Embedding (tSNE) map derived from flow cytometric analysis indicated the populations of the infiltrating immune cells, including CD3^+^ T cells (CD45^+^CD3^+^), natural killer (NK) cells (CD45^+^CD3^−^CD56^+^), natural killer T (NKT) cells (CD45^+^CD3^+^CD56^+^), monocytes (CD45^+^CD14^+^), macrophages (CD45^+^CD14^+^CD68^+^), and myeloid-derived suppressor cells (MDSC) (CD45^+^CD11b^+^CD33^+^). Percentages of immune cell populations within CD45^+^ cells are shown on the bottom panel (*n* = 32). **b** PD-L1 expression profiles detected by flow cytometry in HCC tissues with high or low GOLM1 expression. tSNE map derived from flow cytometric analysis indicated the intensity of PD-L1 expression in CD45^+^ cells. Mean fluorescence intensity (MFI) of PD-L1 staining in tumor and stromal cells (CD45^−^), monocytes (CD45^+^CD14^+^), macrophages (CD45^+^CD14^+^CD68^+^) is shown on the bottom panel (*n* = 32). **c** Representative tSNE map derived from flow cytometric analysis of CD8^+^ T cells in HCC tissues with high or low GOLM1 expression. **d** The functional state of CD8^+^ T cells was analyzed by flow cytometric quantification of GZMB^+^, IFN-γ^+^, PD-1^+^, TIM3^+^, Ki67^+^, and active-caspase3^+^ cells in HCC tissues with high or low GOLM1 expression (*n* = 32). **e** The differences of CD68 (macrophages), CD8 (CD8^+^ T cells), and PD-L1 levels detected by immunohistochemical staining (IHC) in human HCC tissues with high or low GOLM1. Representative IHC images for GOLM1, CD68, PD-L1, and CD8 expression in HCC tissues with high or low GOLM1 expression (*n* = 60) are shown on the top panel. The statistical analysis of IHC score is shown on the bottom panel. **f** The levels of GOLM1 and PD-L1 were detected by Western blot in HCC tissues and normalized against those of GAPDH using a computerized image system (Image-Pro Plus 6.0). Intensity higher than the median level was defined as high expression. The statistical analysis of quantification is shown on the bottom panel (*n* = 44). *P < 0.05, **P < 0.01, ***P < 0.001, ns: no significant

To evaluate the effects of GOLM1 on HCC immune microenvironment, we examined the immunophenotypes of tumor-infiltrating immune cells with flow cytometry in 32 HCC tissues. The percentages of macrophages and monocytes were significantly higher in GOLM1-high subgroup compared to GOLM1-low subgroup, while a reduction of natural killer (NK) cells was observed in GOLM1-high subgroup (Fig. 1a). No obvious difference was detected in the percentages of other immune cell populations, such as myeloid-derived suppressor cells (MDSCs), regulatory T cells (Tregs), CD4^+^ T cell, CD8^+^ T cell, and natural killer T (NKT) cells (Fig. 1a and Supplementary Fig. S1a). Considering that PD-L1/PD-1 axis contributes to immune suppression in the tumor microenvironment, we detected the expression of PD-L1 on tumor cells and immune cells in HCC tissues. Interestingly, macrophages expressed much higher levels of PD-L1 than tumor cells (CD45^−^ cells) in HCC tissues (Fig. 1b and Supplementary Fig. S1b). This phenomenon was further confirmed in Hepa1-6 subcutaneous and orthotopic tumors in C57BL/6 mice (Supplementary Fig. S1c, d). Moreover, both tumor cells and macrophages expressed higher levels of PD-L1 in GOLM1-high subgroup compared to GOLM1-low subgroup (Fig. 1b). These findings suggested that the overexpression of GOLM1 is related to immunosuppressive microenvironment in HCC. The function of CD8^+^ T cells was frequently suppressed in the immunosuppressive microenvironment. To determine the functional status of tumor-infiltrating CD8^+^ T cells, we examined and analyzed inhibitory receptors, cytotoxicity, proliferation, and apoptosis of CD8^+^ T cells with flow cytometry in HCC tissues^22,23^. High expression levels of inhibitory receptors (PD-1 and T-cell immunoglobulin and mucin domain-containing 3 (TIM-3)) and apoptosis (activated caspase 3 (A-caspase3)) but significantly decreased ability to produce effector cytokines (interferon-gamma (IFN-γ) and granzyme B (GZMB)) were found on CD8^+^ T cells in GOLM1-high subgroup compared to GOLM1-low subgroup (Fig. 1c, d). However, no obvious difference was detected in the proliferation (Ki67) of CD8^+^ T cells between GOLM1-low and -high subgroups (Fig. 1d).

To further validate the effect of GOLM1 on macrophages infiltration and PD-L1 expression in HCC tissues, based on the immunostaining scores of GOLM1, the enrolled 60 patients were divided into GOLM1-low (score 0-1) and GOLM1-high (score 2-3) subgroups, and their PD-L1, CD8, and CD68 expressions were evaluated, respectively (Fig. 1e and Supplementary Fig. S1e). Consistent with the above results, a markedly increased level of PD-L1 and abundant CD68^+^ macrophages were observed in HCC with high GOLM1 compared to those with low GOLM1, whereas no significant difference was detected in CD8 expression between the two groups (Fig. 1e). We also used Western blot to analyze the expression levels of GOLM1 and PD-L1 in human HCC tissues from 44 patients. According to the ratio of GOLM1 or PD-L1 to GAPDH, a significantly positive correlation was established between GOLM1 and PD-L1 (Fig. 1f). Taken together, these findings suggested that the overexpression of GOLM1 may contribute to the immunosuppressive microenvironment in HCC and immune-escape of tumor cells.

### GOLM1 promotes TAM infiltration and expression of PD-L1 on both tumor cells and TAMs in HCC

**Fig. 2:**
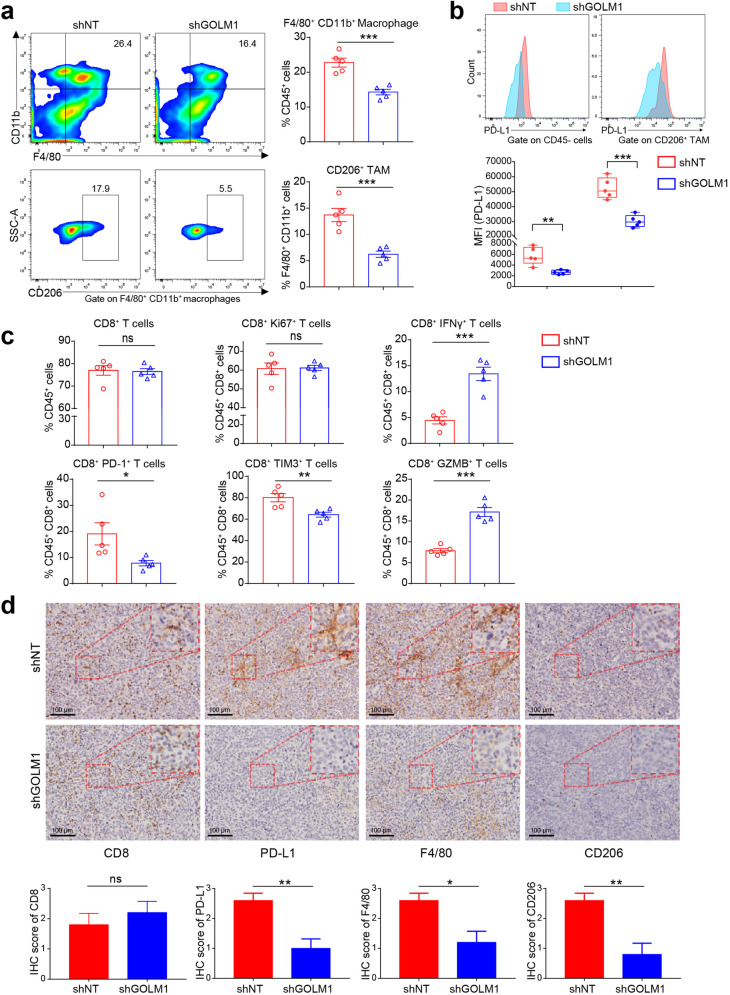
GOLM1 promotes TAM infiltration and PD-L1 expression on TAM in HCC. **a** Flow cytometric analysis of the infiltrated macrophages (F4/80^+^CD11b^+^) and TAMs (F4/80^+^CD11b^+^CD206^+^) in tumor tissues of subcutaneous implantation models of C57BL/6 mice with Hepa1-6-shNT or Hepa1-6-shGOLM1 cells (*n* = 5). **b** Flow cytometric analysis of PD-L1 expression in tumor and stromal cells (CD45^−^), and TAMs (F4/80^+^CD11b^+^CD206^+^) in tumor tissues of subcutaneous implantation models of C57BL/6 mice (*n* = 5). **c** Flow cytometry analysis of the number and functional state of CD8^+^ T cells in the subcutaneous implantation models of C57BL/6 mice. Inhibitory receptors (PD-1 and TIM-3), the ability to produce effector cytokines (IFN-γ and GZMB), and proliferation (Ki67) of CD8^+^ T cells were detected to evaluate the functional state of T cells (*n* = 5). **d** The densities of CD8, PD-L1, F4/80, and CD206 were consistently observed by IHC staining. The quantification is shown on the bottom panel (*n* = 5). *P < 0.05, **P < 0.01, ***P < 0.001, ns: no significant

To determine the effects of GOLM1 on macrophages and PD-L1 expression in vivo in HCC, we established subcutaneous implantation models in C57BL/6 mice using Hepa1-6 transfected with shGOLM1 (mouse, shGOLM1#2) or non-target shRNA control (shNT) (Supplementary Fig. S2a). GOLM1-KD significantly suppressed tumor growth in Hepa1-6 cells. Flow cytometry analysis of macrophages and TAMs infiltration demonstrated that GOLM1-KD significantly decreased the population of macrophages and TAMs but did not alter the population of CD8^+^ T cells and their proliferation (Ki67) significantly, which was consistent with that in human HCC tissues (Fig. 2a and Supplementary Fig. S2b). Furthermore, GOLM1-KD significantly decreased the PD-L1 expression on both tumor cells and TAMs (Fig. 2b). We further evaluated the levels of T cell suppression and activation markers in the CD8^+^ T cell populations with flow cytometry^22,23^. CD8^+^ T cells from Hepa1-6 shGOLM1 tumor exhibited low PD-1 and TIM-3 expression relative to Hepa1-6 shNT tumor, with increased effector cytokines, IFN-γ and GZMB (Fig. 2c and Supplementary Fig. S2b). The densities of PD-L1, CD8, F4/80, and CD206 were consistently observed by immunohistochemistry (IHC) staining (Fig. 2d). Consistent with the above results, a decreased level of TAM infiltration and PD-L1 expression was observed in Hepa1-6 shGOLM1 tumor, whereas no significant difference was detected in CD8 expression between the two groups. Collectively, these findings indicated that GOLM1 promotes the TAM infiltration and expression of PD-L1 on both HCC cells and TAMs in the tumor microenvironment, which induces T cell suppression in HCC.

### GOLM1 upregulates expression of PD-L1 on HCC cells via CSN5-mediated PD-L1 deubiquitination

**Fig. 3:**
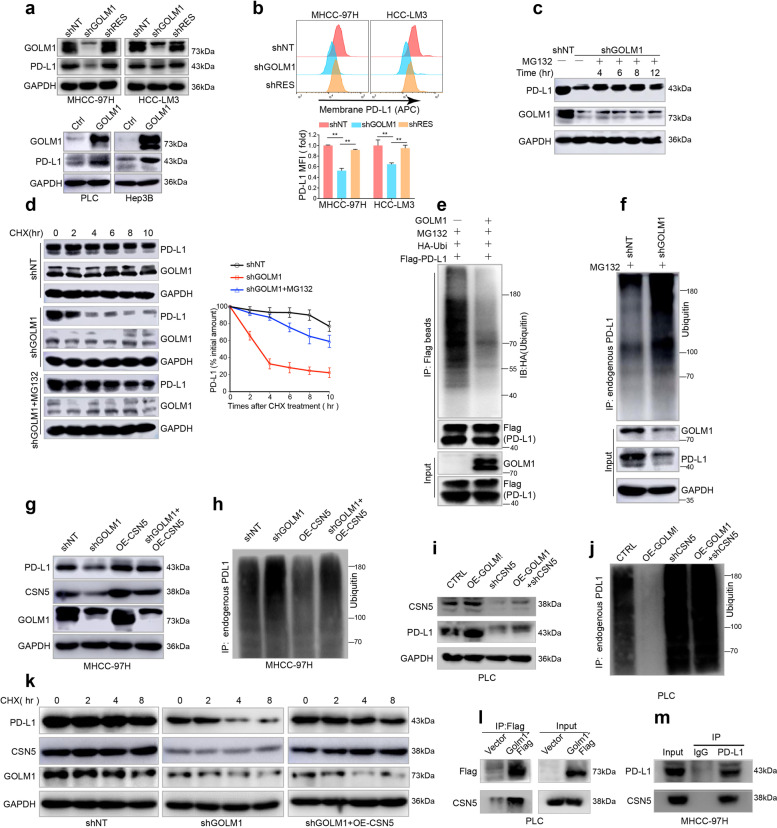
GOLM1 upregulates PD-L1 expression via CSN5-mediated PD-L1 deubiquitination in HCC cells. **a** The GOLM1 expression levels and the corresponding PD-L1 levels in HCC cells detected by Western blot when GOLM1 was knocked down by shGOLM1 and then restored by shRES-GOLM1 in MHCC-97H and HCC-LM3 cells (top panel), and when GOLM1 was exogenously upregulated by transfection of GOLM1^FLAG^ in PLC and Hep3B cells (bottom panel). **b** Flow cytometric analyses of membrane PD-L1 expression in MHCC-97H and HCC-LM3 cells after downregulation and rescue of GOLM1 expression. The relative quantification is shown on the bottom panel. **c** Immunoblot showed that GOLM1-KD significantly decreased PD-L1 expression in MHCC-97H cells, which was alleviated by the proteasome inhibitor MG132 (10 μM). **d** Protein stability assay showed that GOLM1-KD significantly accelerated the degradation of PD-L1 protein, which could be attenuated by MG132. HCC cells were treated with 20 μg/mL cycloheximide (CHX) at indicated intervals and subjected to Western blot (left panel). The quantification is shown on the right panel. Ubiquitination assay of PD-L1 in HEK293T cells (**e**) and MHCC-97H-shGOLM1 cells (**f**) transfected with the indicated plasmids. Ubiquitinated PD-L1 was immunoprecipitated and subjected to Western blot analysis with an antibody against ubiquitin. Cells were treated with MG132 before ubiquitination analysis. Western blot analysis demonstrated that PD-L1 decrease (**g**) and ubiquitination (**h**) induced by GOLM1 knockdown were abolished by overexpression of CSN5. Western blot analysis demonstrated that upregulated expression (**i**) and decreased ubiquitination (**j**) of PD-L1 induced by GOLM1 overexpression was abolished by knockdown of CSN5. **k** Western blot analysis demonstrated that PD-L1 protein degradation induced by GOLM1 knockdown was abolished by overexpression of CSN5. **l** Co-IP determined the interaction of GOLM1 and CSN5 from PLC cells transfected with Flag-GOLM1. **m** The interaction of endogenous PD-L1 with CSN5 in MHCC-97H cells was validated by Co-IP

To further evaluate the functional roles of GOLM1 on the PD-L1 level of HCC cells, we generated three GOLM1-specific short hairpin RNAs (shRNAs) mediated by lentivirus to knockdown (KD) GOLM1 in HCC cells (MHCC-97H and HCC-LM3 cells), shGOLM1#3, which induced the most significant knockdown effect, was adopted for further studies. GOLM1-KD induced by shGOLM1 resulted in a significant decrease of PD-L1 in MHCC-97H and HCC-LM3 cells (Fig. 3a). Then, we reintroduced the recombinant GOLM1 that was not sensitive to shRNA (shRES-GOLM1) into the GOLM1-KD cells and found that the re-expression of GOLM1 rescues the decreased PD-L1 expression in GOLM1-KD HCC cells, thereby excluding the possibility of off-target effects. On the other hand, exogenous upregulation of GOLM1 by transfection of GOLM1^FLAG^ significantly elevated the PD-L1 level in Hep3B and PLC cells with low intrinsic GOLM1 (Fig. 3a and Supplementary Fig. S3a). Owing to the critical role of membrane PD-L1 in tumor immune-escape, we detected the PD-L1 level of cytomembrane using flow cytometry and found similar changes in HCC cells after downregulation of GOLM1 (Fig. 3b). These results indicated that GOLM1 is critical in upregulating PD-L1 expression in HCC cells.

An increase in PD-L1 expression results from activated PD-L1 transcription and/or enhanced PD-L1 protein stability or recycling^24–26^. First, we found that knocking down GOLM1 has no significant effect on PD-L1 mRNA expression (Supplementary Fig. S3b) in MHCC-97H or HCC-LM3 cells, implying that the regulation is not at the transcriptional level. To explore whether PD-L1 was regulated by GOLM1 through endocytosis and recycling of PD-L1, we performed immunofluorescence analysis and did not observe significant co-localization of PD-L1 with early endosome antigen 1 (EEA1), Rab7, Rab11, lysosomal associated membrane protein 1 (Lamp1), or transferrin receptor (TFRC) (Supplementary Fig. S3c). These findings indicate that PD-L1 increase induced by GOLM is not through enhanced endocytosis and recycling of PD-L1^25^. In addition, we found that the proteasome inhibitor MG132 could alleviate the downregulation of PD-L1 by GOLM1-KD in MHCC-97H cells (Fig. 3c), but the lysosomal inhibitors chloroquine (CQ) and NH_4_Cl did not display an obvious influence on the PD-L1 protein level^26^ (Supplementary Fig. S3d). The protein stability assay showed that GOLM1-KD significantly accelerated the degradation of PD-L1 protein, which could be attenuated by MG132 (Fig. 3d). These results suggested that GOLM1 upregulates PD-L1 protein expression in a proteasome-dependent manner.

To further elucidate whether GOLM1 deubiquitinates PD-L1 to increase protein stabilization, transfection of PD-L1 alone in HEK293T was demonstrated to increase PD-L1 poly-ubiquitination, whereas co-transfection of GOLM1 with PD-L1 causes an obvious decrease of PD-L1 poly-ubiquitination (Fig. 3e). On the other hand, GOLM1-KD caused a significant increase in PD-L1 poly-ubiquitination in MHCC-97H cells (Fig. 3f). Furthermore, CSN5 is a deubiquitinating enzyme that upregulates PD-L1 protein stability^24^, and hence, we explored whether GOLM1 stabilizes PD-L1 via CSN5-mediated PD-L1 deubiquitination. We generated two CSN5-specific shRNAs to knockdown CSN5 in PLC cells. CSN5-KD induced by shCSN5 resulted in a significant decrease of PD-L1 expression and an increase of PD-L1 ubiquitination in PLC cells (Supplementary Fig. S3e–f). Then, the results demonstrated that PD-L1 ubiquitination and downregulation of PD-L1 induced by GOLM1-KD was abolished by CSN5 overexpression (Fig. 3g, h). And upregulated expression and decreased ubiquitination of PD-L1 induced by GOLM1 overexpression was abolished by knockdown of CSN5 (Fig. 3i, j). The cycloheximide (CHX) assay demonstrated rapid degradation of PD-L1 in GOLM1-KD MHCC-97H cells, which could be blocked by CSN5 overexpression (Fig. 3k). Moreover, the interaction among GOLM1, CSN5 and PD-L1 was verified with co-immunoprecipitation (Co-IP) assays in PLC cells transfected with Flag-GOLM1 (Fig. 3l) and in MHCC-97H cells (Fig. 3m). Together, these data suggested that GOLM1 upregulates PD-L1 by promoting CSN5-mediated deubiquitination and stabilization of PD-L1 in HCC cells.

### GOLM1 increases exosomal PD-L1 levels without influence on exosome production from HCC cells

Although we have shown that GOLM1 upregulated expression of PD-L1 on HCC cells in vitro, it is confusing that tumor cells still expressed much lower levels of PD-L1 than macrophages in human HCC tissues and in vivo. Recent reports have indicated that PD-L1 exists in exosomes and exosomes can transport PD-L1 to other cell types in the tumor microenvironment^20,27–29^. Since GOLM1 serves as a specific cargo adaptor mediating transport processes between trans-Golgi network and plasma membrane^21^, we hypothesized that in addition to promoting the expression of PD-L1 on HCC cells, GOLM1 might facilitate the transfer of PD-L1 from tumor cells to macrophages via exosomes in the HCC microenvironment.

**Fig. 4:**
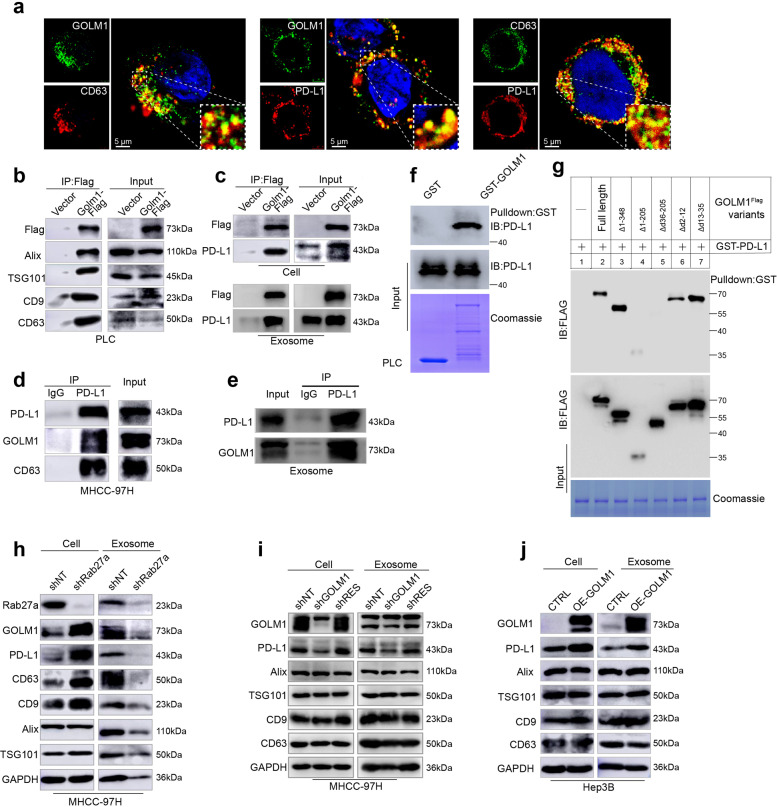
GOLM1 increases exosomal PD-L1 levels from HCC cells. **a** Immunofluorescence staining showed a co-localization of GOLM1 with exosome marker CD63 (left panel) or PD-L1 (middle panel) and CD63 with PD-L1 (right panel) in the multivesicular endosomes (MVEs), which are the precursor forms of exosomes before released. **b** Co-IP determined the association of GOLM1 and exosome markers CD63, CD9, TSG101, and Alix from PLC cells transfected with Flag-GOLM1. **c** Co-IP demonstrated the association of exogenous GOLM1 with PDL-L1 in GOLM1^Flag^ PLC cells and exosomes: PLC cells transfected with FLAG-GOLM1 or empty vector (control) were subjected to IP using anti-FLAG magnetic beads. **d** The association of endogenous PD-L1 with GOLM1 and CD63 in MHCC-97H cells was validated. **e** The association of endogenous PD-L1 with GOLM1 in MHCC-97H exosomes was validated. **f** GST pulldown assays showed a direct interaction between GOLM1 and PD-L1. GST- GOLM1 was expressed in *E. coli* and purified. Full-length PD-L1 was produced via in vitro translation. **g** Mapping the regions of GOLM1 that interact with PD-L1. Various FLAG-tagged truncated GOLM1 were prepared to test the interaction with GST-PD-L1, GOLM1 was detected by anti-Flag antibody after GST pulldown. Coomassie blue staining of one-tenth of the amount of each GST-PD-L1 fragment. **h** Western blotting analysis showed after Rab27a was knocked down, intracellular accumulation of PD-L1 and GOLM1 was detected in MHCC-97H cells, whereas PD-L1 and GOLM1 in exosomes decreased significantly, and the abundance of exosomes, indicated by exosomal markers, ALIX, TSG101, CD63, and CD9, was inhibited significantly. **i**, **j** Western blot analysis of the exosomes isolated from an equivalent number (6 × 10^7^) of HCC cells indicating that GOLM1 facilitates the secretion of exosomal PD-L1. MHCC-97H cells with endogenous high-GOLM1 (**i**) and low-GOLM1 Hep3B cells (**j**) were used

Herein, we purified exosomes from the culture supernatant of MHCC-97H cells, and verified them by transmission electron microscopy and nanoparticle tracking analysis (Supplementary Fig. S4a, b). Most of the isolated vesicles were 30–150 nm, the typical size of exosomes. Exosomes were further verified by Western blot analyses of the exosomal markers, CD63, ALIX, TSG101, and CD9 (Supplementary Fig. S4c). However, calnexin, an endoplasmic reticulum membrane protein, was not detected in exosome samples (Supplementary Fig. S4c). Furthermore, immunofluorescence staining revealed a co-localization of GOLM1 and PD-L1 with CD63 in the multivesicular endosomes (MVEs), which are the precursors of exosomes before released (Fig. 4a). Moreover, GOLM1 was also found to co-localize with PD-L1 in the MVEs (Fig. 4a). The association of GOLM1 with exosome markers CD63, CD9, TSG101, and Alix was verified with co-immunoprecipitation (Co-IP) assays in PLC cells transfected with Flag-GOLM1 (Fig. 4b). In addition, the Co-IP of PD-L1 with GOLM1 and CD63 was also validated in GOLM1^Flag^ PLC cells and MHCC-97H cells (Fig. 4c, d), respectively. Moreover, the interaction of exosomal GOLM1 and PD-L1 was also validated in GOLM1^Flag^ PLC cells and MHCC-97H cells ((Fig. 4c, e).

To further examine whether GOLM1 interacts directly with PD-L1, GST- GOLM1 was expressed in *Escherichia coli* and purified. The full-length PD-L1 was produced via in vitro translation. GST pulldown assays indicated that GOLM1 directly interacts with PD-L1 (Fig. 4f). To determine the regions mediating the interaction, various FLAG-tagged truncated GOLM1 constructs were prepared to test the interaction with GST-PD-L1 individually. GST pulldown assays indicated that the region spanning residues 36–205 of GOLM1 was responsible for the interaction with GST-PD-L1 (Fig. 4g).

In order to clarify whether exosome secretion influences the abundance of PD-L1 in HCC cells, Rab27a^30^, mediating exosomes release, was knocked down, which significantly inhibited the secretion of exosomes as measured by exosomes markers, ALIX, TSG101, CD63, and CD9, or the total amount of exosomal protein (Fig. 4h and Supplementary Fig. S4d). The results showed that the intracellular accumulation of GOLM1 and PD-L1 in Rab27a-KD MHCC-97H cells and GOLM1 and PD-L1 decreased significantly in exosomes (Fig. 4h). This indicated that exosome secretion reduces the abundance of PD-L1 in HCC cells. To further investigate the effect of GOLM1 on the secretion of exosomal PD-L1, we isolated exosomes from an equal number of MHCC-97H-shGOLM1, MHCC-97H-shNT, and MHCC-97H-shRES-GOLM1 cells. Western blot analysis showed that GOLM1 did not affect the exosome amount as indicated by exosomal markers or the nanoparticle tracking analysis (Supplementary Fig. S4e), whereas the level of PD-L1 in exosomes decreased markedly in MHCC-97H-shGOLM1 cells and was rescued when reintroducing shRES-GOLM1 into GOLM1-KD cells (Fig. 4i). Similarly, exosomal PD-L1 increased in Hep3B-GOLM1^Flag^ cells with no change in the exosome abundance (Fig. 4j). Collectively, these results suggested that GOLM1 significantly upregulates the exosomal PD-L1 level but has no obvious effect on the exosome amount derived from HCC cells.

### GOLM1 facilitates PD-L1 sorting into exosomes via suppressing Rab27b in trans-Golgi network area

**Fig. 5:**
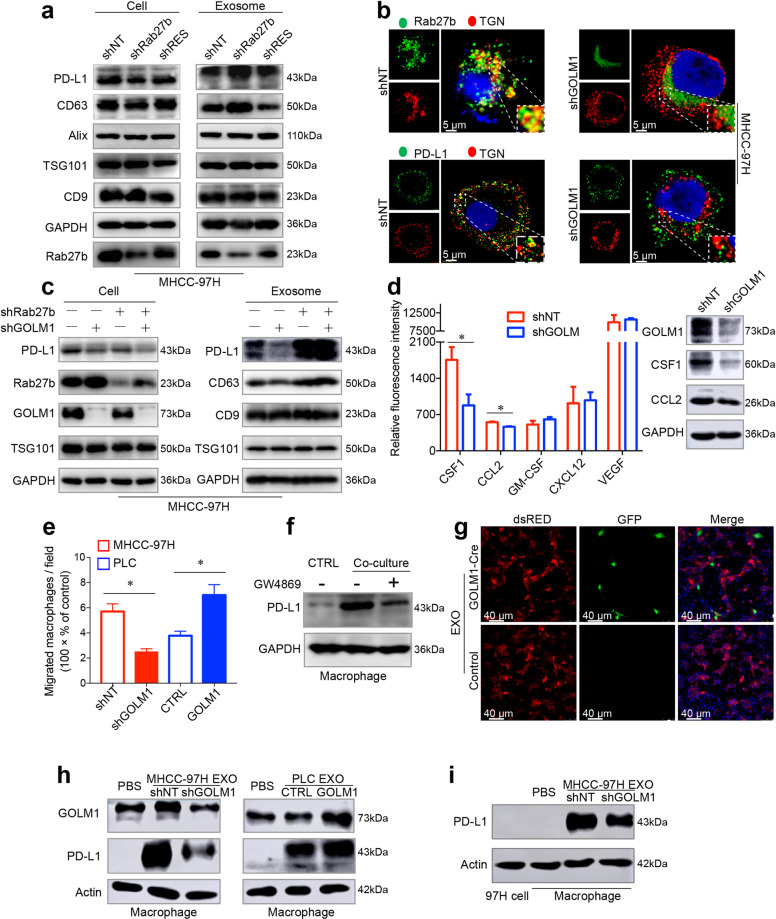
GOLM1 facilitates PD-L1 sorting into exosomes and transport to macrophages via suppression of Rab27b. **a** Western blot analysis showed the alterations of PD-L1 expression on HCC cells and in exosomes from MHCC-97H cells after knockdown of Rab27b and reintroducing shRES-Rab27b into Rab27b-KD cells. **b** Immunofluorescence images demonstrated the co-localization of Rab27b (top panel) or PD-L1 (bottom panel) with TGN marker TGN46 in MHCC-97H-shNT and MHCC-97H-shGOLM1 cells. **c** Western blotting showed that the knockdown of Rab27b significantly increased the reduced PD-L1 expression in exosomes induced by GOLM1-KD in MHCC-97H cells. **d** Western blot validated the screening results of an antibody microarray containing a panel of 42 chemokines related to the chemotaxis of TAMs, in the supernatant of MHCC-97H-shGOLM1 and MHCC-97H-shNT cells. **e** Migration assays revealed the association of macrophage migration with GOLM1 levels in HCC cells. **f** Western blotting analysis showed PD-L1 expression on macrophages after co-culture with MHCC-97H treated with exosome secretion inhibitor GW4869 (20μM) or DMSO (control) for 48 h. **g** Immunofluorescence images of THP1-derived macrophages. Exosomes isolated from MHCC-97H-GOLM1-Cre cells were added to cultures of macrophages expressing LoxP-DsRed-Stop-LoxP-eGFP. Macrophages switched from red to green indicated that GOLM1-Cre protein was successfully transferred by exosomes into macrophages. **h** Western blot showed the alterations in PD-L1 levels of macrophages co-cultured with exosomes derived from HCC cells with various GOLM1 expressions. **i** Western blot showed the PD-L1 levels of MHCC-97H cells and macrophages co-cultured with exosomes derived from MHCC-97H cells. *P < 0.05

As a Golgi-related protein, GOLM1 interacts with Rab family, which controls different steps of vesicular trafficking^31^. To further explore the mechanism of GOLM1 facilitating PD-L1 to sort into exosome, we investigated whether the two Rab27 isoforms, Rab27a and Rab27b, are involved in exosome secretion pathway^32^ and found that Rab27a-KD, rather than Rab27b-KD, induced a significant decrease in exosomes in MHCC-97H cells (Fig. 4g). However, after GOLM1 knockdown, Rab27b, but not Rab27a, was significantly increased in HCC cells (Supplementary Fig. S4f). Moreover, other Rab family proteins, such as Rab5, Rab7, and Rab11, involved in vesicular cargo transport, were not affected when GOLM1 was knocked down (Supplementary Fig. S4f). In addition, knockdown of Rab5 decreased PD-L1 expression on HCC cell surface but dramatically increased the levels of exosomal PD-L1 and marker proteins of exosomes (Supplementary Fig. S4g). Knockdown of Rab7 induced an obvious decrease in the levels of PD-L1 and exosomal marker proteins both on the cell surface and in exosomes (Supplementary Fig. S4h), while the knockdown of Rab11 did not influence the exosomal PD-L1 (Supplementary Fig. S4i). However, the knockdown of Rab27b dramatically reduced PD-L1 expression on the HCC cell surface but resulted in an increase in PD-L1 level in exosomes without an obvious change in the abundance of exosomes from HCC cells (Fig. 5a). These effects were neutralized by reintroducing shRES-Rab27b into Rab27b-KD cells. Also, Rab27b was concentrated in the trans-Golgi network (TGN) area in MHCC-97H-shNT cells compared to MHCC-97H-shGOLM1 cells (Fig. 5b). These findings indicate that Rab27b is required for the transportation of PD-L1 to the plasma membrane and suppression of Rab27b diverts PD-L1 into endosomes in the TGN area, thus increasing exosomal PD-L1 levels.

Next, to investigate whether GOLM1 promotes PD-L1 sorting into exosomes through downregulating Rab27b expression, we knocked down Rab27b in GOLM1-KD MHCC-97H cells. The knockdown of Rab27b significantly increased the decline in PD-L1 expression in exosomes induced by GOLM1-KD in MHCC-97H cells (Fig. 5c). Moreover, immunofluorescence staining demonstrated that GOLM1-KD decreased the PD-L1 co-localization with TGN in MHCC-97H cells (Fig. 5b). Taken together, these findings indicated that GOLM1 facilitates PD-L1 sorting into exosomes by suppressing Rab27b in the TGN area.

### GOLM1 upregulates the expression of PD-L1 on macrophages via facilitating the transport of exosomal PD-L1 from HCC cells

To explain the above findings that overexpression of GOLM1 increases the TAM infiltration in HCC, we surveyed the changes of a panel of 42 chemokines related to the chemotaxis of TAM using an antibody microarray (Fig. 5d and Supplementary Fig. S5a). The concentrations of colony-stimulating factor 1 (CSF1) and C-C motif chemokine ligand 2 (CCL2) in MHCC-97H-shGOLM1 cells were significantly decreased compared to that of the controls (Fig. 5d). Furthermore, we also evaluated HCC cells with various GOLM1 levels on the migration of macrophages and found that the supernatants of MHCC-97H cells with high endogenous GOLM or PLC cells transfected with GOLM1 plasmid significantly increased the migration ability of macrophages (Fig. 5e and Supplementary Fig. S5b). These observations might partially explain the alterations in TAM infiltration. Additionally, a high PD-L1 expression was detected in macrophages when co-cultured with MHCC-97H cells, while decreased PD-L1 expression was observed when macrophages were treated with exosome secretion inhibitor GW4869 (Fig. 5f). Macrophages cultured with cytokines CSF1 and CCL2 didn’t increase the PD-L1 level in vitro (Supplementary Fig. S5c).

This indicated that exosome may modulate PD-L1 expression on macrophages in HCC cells.

To investigate the underlying mechanism, we utilized the Cre-LoxP system^33,34^ and evaluated the delivery of exosomal cargo from HCC cells into macrophages (Supplementary Fig. S5d). Exosomes were isolated from MHCC-97H cells transfected with GOLM1-Cre and analyzed by Western blot (Supplementary Fig. S5e). Then, we transfected macrophages (derived from THP1 cells) with pLV-CMV-LoxP-DsRed-Stop-LoxP-eGFP virus. After transfection and selection, the fluorescence of macrophages was detected in the red, but not the green channel (Supplementary Fig. S5f). Next, we directly added exosomes isolated from MHCC-97H-GOLM1-Cre cells to the macrophages-LoxP-DsRed-Stop-LoxP-eGFP and observed that the selected macrophages switched from red to green (Fig. 5g), indicating that the exosomes carrying GOLM1-Cre were taken up by these macrophages and the exosomal cargo worked within the macrophages. Western blot analysis showed that the macrophages treated with exosomes from MHCC-97H cells had significantly increased levels of PD-L1 (Fig. 5h) and M2 macrophages marker mannose receptor C-type 1 (CD206) but a decreased level of M1 macrophages marker nitric oxide synthase 2 (iNOS) (Supplementary Fig. S5g). GOLM1-KD significantly attenuated the upregulation of PD-L1 on macrophages induced by exosomes derived from MHCC-97H cells (Fig. 5h). Moreover, after co-cultured with exosomes derived from MHCC-97H cells, macrophages expressed much higher PD-L1 than HCC cells (including MHCC-97H-shNT and MHCC-97H-shGOLM1 cells) (Fig. 5i), corresponding to the results in vivo. Collectively, these data indicated that GOLM1 upregulates the expression of PD-L1 on macrophages via facilitating the transport of exosomal PD-L1 from HCC cells to macrophages.

### TAM inhibitor and anti-PD-L1 combination neutralizes T cell suppression and inhibits tumor growth in HCC

**Fig. 6:**
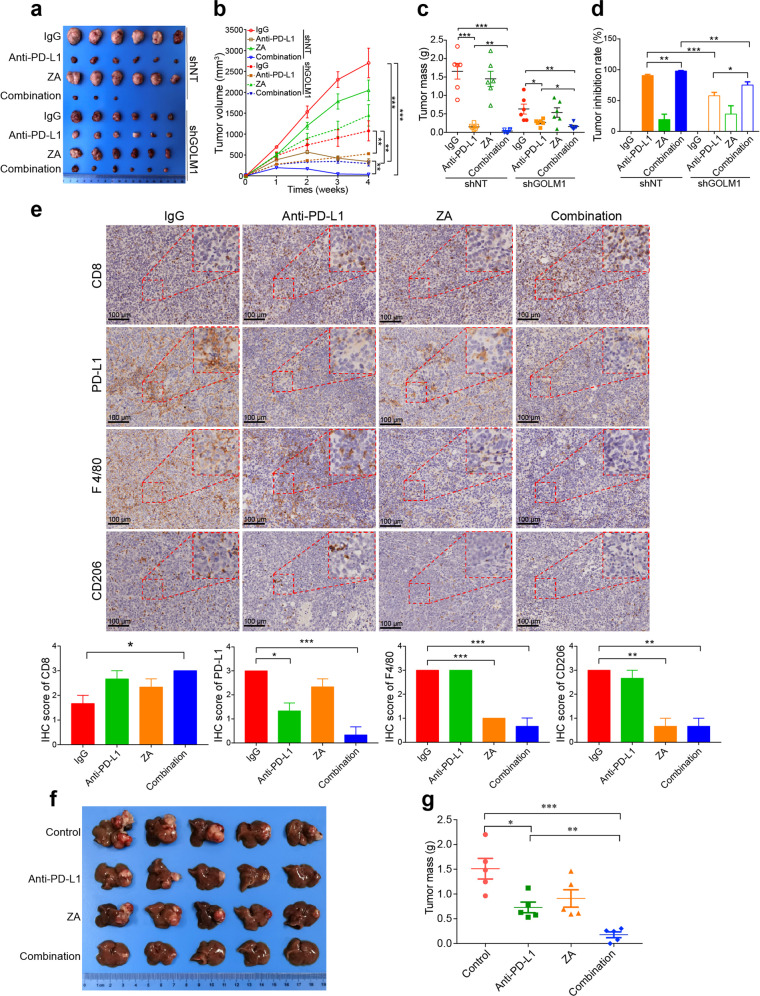
Inhibition of TAM infiltration enhances the efficacy of anti-PD-L1 immunotherapy in HCC mouse model. **a**–**d** The inhibitory effects of anti-PD-L1 Ab on tumor growth in subcutaneous implantation C57BL/6 mouse model using Hepa1-6 shNT or Hepa1-6 shGOLM1 cells. When tumor size was 40 mm^3^, mice were treated with IgG isotype control, anti-PD-L1 antibody (200 μg i.p. injection every 3 days), ZA (100 μg/kg i.p. injection every 3 days), and anti-PD-L1 antibody in combination with ZA until the treatment endpoint. Representative images of subcutaneous tumors (**a**), tumor growth curves (**b**), tumor weight (**c**), and tumor inhibition rate (**d**) for indicative groups (*n* = 6 mice/group). **e** Representative images and quantification of IHC staining of PD-L1, CD8, F4/80, and CD206 in hepa1-6 shNT subcutaneous tumors (*n* = 3 per group). **f**, **g** Representative images and tumor weight analysis in orthotopic implantation models of Hepa1-6 shNT (*n* = 5 mice/group). *P < 0.05, **P < 0.01, ***P < 0.001, ns: no significant

Since the above findings suggested that GOLM1 upregulates PD-L1 expression on macrophages and limits the anti-tumor T cell response, we sought to determine whether inhibiting TAM infiltration combined with anti-PD-L1 immunotherapy relieves T cell suppression and inhibits tumor growth. Zoledronic acid (ZA) is a clinically available drug for bone metastases by targeting osteoclasts^35,36^ and is also an inhibitor of macrophages. We first established subcutaneous implantation mouse models using Hepa1-6-shNT or Hepa1-6-shGOLM1 cells and divided them into four groups that were administered IgG, ZA, PD-L1 antibody, and the combination of PD-L1 antibody and ZA, respectively. The tumor growth and immune cell infiltration of these cells were monitored. The combination therapy impaired tumor growth and reduced tumor burden more significantly than ZA or anti-PD-L1 alone in mice bearing Hepa1-6 shNT (Fig. 6a–d). Moreover, the combination therapy increased the infiltration of CD8^+^ T cells in tumor (Fig. 6e). Both ZA alone and in combination with anti-PD-L1 substantially decreased TAM infiltration in tumors of the Hepa1-6 shNT mice (Fig. 6e). However, this synergistic suppression effect of the combination therapy on HCC growth was weaken in the mouse models bearing Hepa1-6-shGOLM1 cells, demonstrating that HCC with high expression of GOLM1 is sensitive to the combination therapy (Fig. 6a–d). These findings were further validated in orthotopic implantation models of Hepa1-6 cells (Fig. 6f, g), which showed that macrophage inhibitor enhances the therapeutic effect of anti-PD-L1 in HCC, especially those with GOLM1 overexpression.

**Fig. 7:**
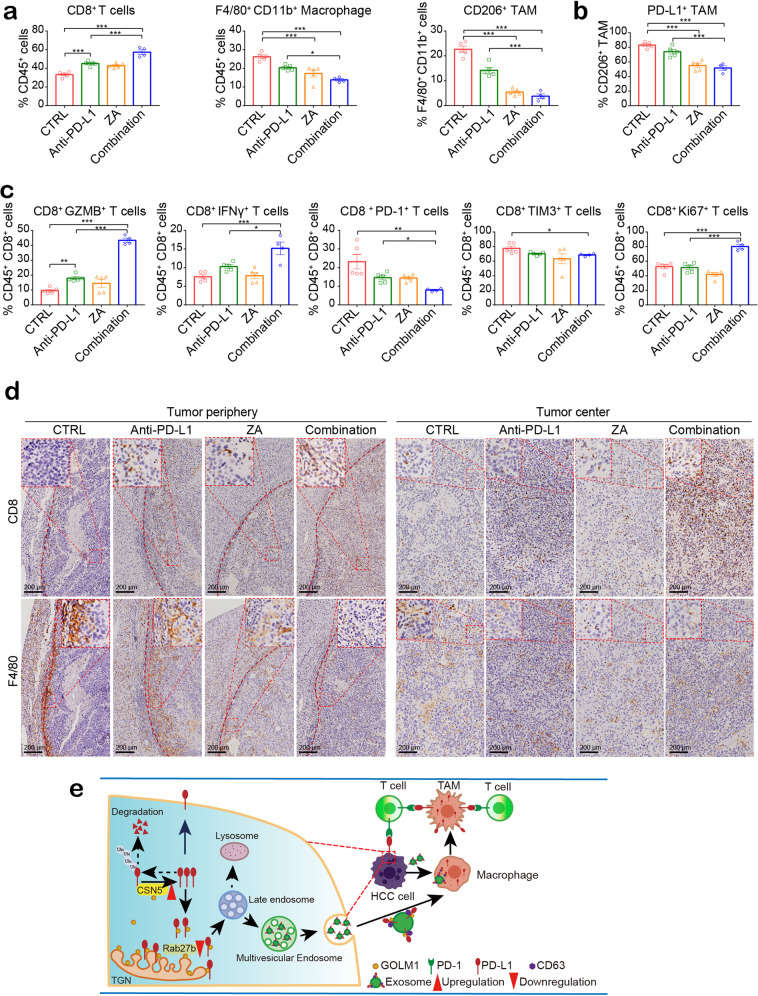
TAM inhibitor and anti-PD-L1 combination decreases PD-L1+ TAMs infiltration and neutralizes T cell suppression. **a**, **b** Flow cytometric quantification of CD8^+^ T cells, F4/80^+^ CD11b^+^ (macrophages), and F4/80^+^ CD11b^+^ CD206^+^ (TAMs), as well as PD-L1^+^ TAMs in tumor tissues from orthotopic implantation models of Hepa1-6 cell in C57BL/6 mice. ZA and anti-PD-L1 combination increased the infiltration of CD8^+^ T cells and decreased the infiltration of TAMs (**a**) with a significantly decreased percentage of PD-L1^+^ TAMs (**b**). **c** The functional state of T cells was analyzed by flow cytometric quantification of GZMB^+^, IFN-γ^+^, PD-1^+^, TIM3^+^, and Ki67^+^ cells among CD8^+^ T cells in HCC tissues from indicative groups (*n* = 5 mice in Control, anti-PD-L1, and ZA groups, *n* = 4 mice in combination group). **d** IHC data suggested that in the combination treatment group, infiltration of TAMs decreased sharply in the peripheral tumor region, whereas CD8^+^ T cell increased significantly in the central tumor region. Representative immunohistochemical staining images of CD8 and F4/80 in tumor periphery and center are shown. **e** A schematic diagram describing the molecular mechanisms of the immunosuppressive microenvironment drove by GOLM1 in HCC. *P < 0.05, **P < 0.01, ***P < 0.001, ns: no significant

In order to understand the synergistic mechanism induced by ZA and anti-PD-L1 combination, we analyzed the number and functional state of CD8^+^ T cells and TAMs in the orthotopic Hepa1-6 HCC model. Flow cytometric analyses revealed a significant reduction in the number of TAMs, a significantly decreased percentage of PD-L1^+^ TAMs, and increased infiltrating CD8^+^ T cells in mice treated with the combination therapy as compared to control mice or mice receiving ZA or anti-PD-L1 alone (Fig. 7a, b and Supplementary Fig. S6a, b). Moreover, the effector cytokines, IFN-γ and GzmB, in CD8^+^ T cells increased significantly in the combination treatment group with higher CD8^+^ T cell proliferation (Ki67) in tumors compared to that of ZA or anti-PD-L1 alone (Fig. 7c and Supplementary Fig. S6b). In addition, the combination treatment significantly decreased the expression of inhibitory receptors, PD-1 and TIM-3, on CD8^+^ T cells (Fig. 7c and Supplementary Fig. S6b). Consistently, IHC staining showed that the infiltration of TAMs was decreased in the tumor periphery of mice administered ZA alone or in combination with anti-PD-L1 (Fig. 7d). Furthermore, the combination therapy also increased the CD8^+^ T cell infiltration in the tumor region (Fig. 7d), especially enrichment in the central area of the tumor. These results indicated that the combination treatment alleviates CD8^+^ T cell suppression and improves the response to anti-PD-L1 therapy in HCC by inhibiting PD-L1^+^ TAMs infiltration.

Taken together, these findings determine a close correlation between GOLM1 and immunosuppression. GOLM1 upregulates PD-L1 expression via CSN5-mediated PD-L1 deubiquitination in HCC cells. Furthermore, GOLM1 facilitates PD-L1 sorting into exosomes via suppressing Rab27b in HCC cells, which upregulates the PD-L1 expression of macrophages through the transport of exosomal PD-L1 to macrophages. TAM inhibitor combined with anti-PD-L1 therapy depletes PD-L1^+^ TAMs infiltration and neutralizes CD8^+^ T cell suppression in HCC. Schematic diagram of the molecular mechanism was shown in Fig. 7e.

## Discussion

As one component of the Golgi complex, GOLM1 plays a key role in sorting and modification of cargo proteins in the rough endoplasmic reticulum and protein transportation through the Golgi apparatus. Upregulation of GOLM1 has been observed in response to viral infection^37^. GOLM1 is overexpressed in HCC, lung cancer, prostate cancer and is a serum biomarker of HCC^38–40^. GOLM1-enriched exosomes accelerated GOLM1-poor cells proliferation and migration by activating glycogen synthase kinase-3b/matrix metalloproteinases (GSK-3b/MMPs) signaling axis of recipient cells in HCC^41^. GOLM1 expression can be modulated by the mammalian target of rapamycin (mTOR) signaling pathway and blocked with mTOR inhibitor rapamycin in HCC^42^. Deoxycholic acid can upregulate the expression and release of GOLM1 via activating the NF-κB pathway and destroying the Golgi structure in liver disease^43^. Moreover, in bone marrow mesenchymal stem cells, GOLM1 promotes glutamine metabolism via activation of mTOR signaling pathway^44^. In the current study, we demonstrated that in addition to the effects on cancer cells, the overexpression of GOLM1 also influences the tumor microenvironment and response to ICB of HCC. First, we evaluated the immunophenotypes of tumor-infiltrating immune and PD-L1 expression profile in HCC with various GOLM1 levels and found that the overexpression of GOLM1 is associated with TAM infiltration and decreased NK cells in HCC tissues. The level of PD-L1 is much higher in HCC with a high GOLM1 level, especially expressed on the infiltrating TAMs.

Additionally, we evaluated the functional states of CD8^+^ T cells in HCC with various GOLM1 levels and found that although no obvious difference was detected in the number and proliferation of CD8^+^ T cells between GOLM1-low and -high subgroups, their functional states were varied with decreased levels of effector cytokines (IFN- γ and GZMB) and increased expression of inhibitory receptors (PD-1 and TIM-3) in CD8^+^ T cells from GOLM1-high HCC. These findings suggested that the overexpression of GOLM1 in HCC cells is related to T cell suppression. In order to further validate this finding, we used subcutaneous and orthotopic implantation mouse models to perform gain- and loss-of-function analyses and provide evidence to support that GOLM1 promotes the expression of PD-L1 both on HCC cells and TAMs, and induces T cell suppression in HCC.

Next, we firstly explored the mechanism underlying GOLM1-regulated expression of PD-L1 on HCC cells. After excluding the possibilities of the transcriptional pathway, enhanced lysosomal degradation, and recycling, we found that GOLM1-KD significantly accelerates the degradation of PD-L1 protein, and thus, decreases the PD-L1 level in HCC cells, which could be attenuated by MG132, a proteasome inhibitor. These phenomena suggested that GOLM1 upregulates PD-L1 protein expression by maintaining the stability of PD-L1 in a proteasome-dependent manner. Moreover, we found that CSN5 overexpression abolishes the downregulation of PD-L1 induced by GOLM1-KD, indicating that GOLM1 promotes PD-L1 stabilization via CSN5-mediated PD-L1 deubiquitination in HCC cells.

Overexpression of PD-L1 facilitates cancer cells to evade anti-tumor immunity. However, it has been reported that cancer cells themselves prefer to maintain a fairly low level of PD-L1^16,45^. In the present study, we found that only a limited subset of HCC cells express high levels of PD-L1, whereas most intratumoral inflammatory cells express higher levels of PD-L1, which is consistent with the previous reports in HCC^16,45^, as well as other kinds of malignancies, such as cholangiocarcinoma, breast cancers, soft tissue sarcoma, and neuroendocrine carcinoma^17,18,46–48^. Macrophages constitute a major component of the infiltrating stromal cells in various malignancies, especially HCC, and our previous study suggested that macrophages in HCC environment are associated with HCC progression, relapse, and poor survival after hepatectomy^49^. In the present study, IHC and flow cytometry demonstrated more infiltration of PD-L1^+^ TAMs in HCC tissues with high GOLM1 expression, and TAMs expressed much higher levels of PD-L1 than tumor cells. Moreover, the co-culture of macrophages with high GOLM1 HCC cells significantly facilitated PD-L1 expression and migration of macrophages. These phenomena indicated that GOLM1, at least partially, upregulates the expression of PD-L1 on macrophages in the tumor microenvironment in addition to cancer cells.

Exosome-mediated interaction between cancer and stromal cells is critical in tumor progression^8,12^. In addition to the surface of cancer cells, PD-L1 can also be found in exosomes^20,27–29^. GOLM1 mediates specific cargo transport between trans-Golgi network and plasma membrane^21^, thereby hinting a putative role of GOLM1 in the regulating sort of PD-L1 into exosomes in HCC cells. In the present study, we utilized gain- and loss-of-function analyses and clarified that GOLM1 significantly upregulates the exosomal PD-L1 level but has no obvious effect on the number of exosomes derived from HCC cells. Furthermore, we provide evidence that GOLM1 interacts directly with PD-L1 in the region spanning residues 36–205 of GOLM1. Additionally, we found that GOLM1 facilitates PD-L1 sorting into exosomes by suppressing Rab27b in the TGN area. Rab family controls vesicular trafficking, including budding, motility, docking, and fusion of different vesicular transport intermediates to acceptor membranes^31^. GOLM1 interacts with Rab family, which could be the potential mechanism of GOLM1 facilitating PD-L1 to sort into the exosomes. Next, we investigated the various members of Rab family involved in vesicular cargo transport and found that Rab27b is required for the transportation of PD-L1 to the plasma membrane, and suppression of Rab27b diverts PD-L1 into the endosomes in the TGN area, thus increasing the exosomal PD-L1 levels. Furthermore, with Cre-LoxP system^33,34^, we demonstrated that HCC cells with GOLM1 overexpression interact with macrophages via exosome and modulate their biological features, including PD-L1 expression, M2 polarization, and migration ability. Collectively, these data indicate that GOLM1 regulates PD-L1 exocytosis by facilitating exosome cargo transport, which results in PD-L1 delivery into macrophages with exosome carrier.

Our results revealed that NK cells were decreased in GOLM1-high subgroup compared to GOLM1-low subgroup. We speculated that one mechanism by which GOLM1 suppresses NK cells is via the upregulation of PD-L1 expressed by TAMs. The interpretation is consistent with a pervious study that PD-L1 expressing TAMs inhibit NK cells activation and cytotoxicity in Hodgkin lymphoma^50^. Another possible mechanism is that GOLM1 mediated CD8^+^ T cell suppression might inhibit NK cells proliferation by reduced secretion of IL-2, which is an important cytokine for NK cell activation and proliferation.

ZA is now clinically available as an anti-metastatic drug that prevents and treats bone metastases in patients with solid tumors by targeting osteoclasts, which are macrophages homing to the bone^51^. It was reported that ZA reduced the numbers of macrophages induced by sorafenib, inhibited tumor progression, and reduced lung metastasis in HCC^52^. Moreover, ZA enhanced the antitumor efficacy of anti-PD-1 therapy in the breast cancer mouse model by decreasing MDSCs^53^. In this study, we found that PD-L1^+^ TAMs depletion enhances the therapeutic effect of anti-PD-L1 in HCC, especially in those with GOLM1 overexpression, through neutralizing CD8^+^ T cell suppression and enhancing antitumor immunity.

In conclusion, the current study revealed that GOLM1 promotes PD-L1 stabilization and facilitates PD-L1 transport into TAMs through exosomes, which results in higher expression of PD-L1 on TAMs than HCC cells and induce CD8^+^ T cells suppression. ZA and anti-PD-L1 combination depletes PD-L1^+^ TAMs infiltration and ameliorates CD8^+^ T cells suppression, indicating the important role of PD-L1^+^ TAM in enhancing the response to anti-PD-L1 immunotherapy. Therefore, targeting PD-L1^+^ TAM could be a novel strategy to enhance the efficacy of ICB in HCC.

## Materials and methods

### Patients and specimens

Fresh HCC tissues were collected from 60 patients who underwent hepatectomy for HCC from January–December 2019 at the Department of General Surgery, Huashan Hospital, Fudan University (Shanghai, China). Clinical samples were collected from patients after obtaining informed consent in accordance with the protocol approved by the Ethics Committee of Huashan Hospital, Fudan University (Shanghai, China).

### Animal models

C57/BL6 mice (male, 6-weeks-old) were obtained from Shanghai Laboratory Animal Co., Ltd (Shanghai, China). Subcutaneous implantation models were established by subcutaneously injecting 5 × 10^6^ Hepa1-6 cells into C57/BL6 mice. Tumor volumes were monitored on the indicated days. For orthotopic implantation models, the subcutaneous tumors of Hepa1-6 were harvested, cut into 1-mm^3^-pieces, and incubated into the left hepatic lobe of C57/BL6 mice. In animal experiments, mice were randomly assigned to each group. For drug intervention, mice were treated with anti-PD-L1 antibody (200 μg intraperitoneal (i.p.) injection every 3 days, rat IgG isotype as control, Bio X Cell, West Lebanon, NH, USA) or/and ZA (100 μg/kg i.p. injection every 3 days, Novartis, Novartis Pharma Schweiz AG, Switzerland) one week after tumor cell inoculation. Then, the animals were sacrificed at a time-defined endpoint, and the tumors were removed, weighed, and processed for flow cytometric analyses. All animal experiments were performed according to state guidelines and approved by the Institutional Animal Care and Use Committee (IACUC) of Fudan University.

### Flow cytometric analysis

Tumor-infiltrating cells were prepared using a gentleMACSTM Dissociator and a tumor dissociation kit (Miltenyi Biotec Inc., Auburn, CA, USA) according to the manufacturer’s instructions. Surface and intracellular flow cytometry staining of isolated cells was performed according to standard protocols and analyzed on a Cytoflex Flow Cytometer (Beckman Coulter Life Sciences). Briefly, cells were incubated in Fc-block before being stained with antibodies. Cells were stimulated with Cell Stimulation Cocktail (eBioscienceTM) for 4 h before staining for intracellular cytokines. For surface staining, cells were washed and stained for 30 min at 4 °C with antibodies. For intracellular staining, the samples were fixated and permeabilized with Cytofix/Cytoperm kit (BD Biosciences) or Transcription Factor Buffer Set (BD Biosciences) as recommended by the manufacturer and stained for 30–45 min at 4 °C with the intracellular antibodies. Dead cells were stained with Fixable Viability Dye 780 (BD Biosciences).

For human tumor tissue, the antibodies used are as follows: anti-CD45-FITC, anti-CD14-Alexa 700, anti-CD68-BV421, anti-CD16-BV510, anti-CD56-BV650, anti-CD19-BV605, anti-CD11B-PE-Cy7, anti-CD33-PE, anti-CD3-PerCP-Cy5.5, anti-CD4-Alexa 700, anti-CD8-BV605, anti-CD25-PE-Cy7, anti-FoxP3-Alexa 647, anti-CD279-PE, anti-CD274-APC, and anti-Ki67-BV421, anti-IFN-Gamma-Alexa 700, anti-TIM-3-BV650,anti-Granzyme B-PE/Cy7,anti-Active Caspase3-Alexa 647.

For mouse tumor tissue, the antibodies used are as follows: anti-CD45-FITC, anti-CD8a-BV510, anti-CD279-APC-R700, anti-TIM3-PE, anti-IFN-Gamma-BV650, anti-Granzyme B-Alexa Fluor 647, anti-CD4-BB700, anti-CD274-BV421, anti-F4/80-PE, anti-CD11b-PE-Cy7, anti-CD11c-BV786, anti-CD206-Alexa 647, and anti-Ki67-PE-Cy7.

To measure cell surface PD-L1 expression, single HCC cells were re-suspended in phosphate-buffered saline (PBS) and stained with primary antibodies APC anti-human PD-L1 (BioLegend) according to standard protocols for flow cytometry. The cells were washed with PBS twice and then were evaluated using a Cytoflex Flow Cytometer (Beckman Coulter Life Sciences). Isotype IgG was used as a negative control. Flow cytometric data were analyzed using the FlowJo software program.

### Isolation and characterization of exosomes

At 90% confluency, the cells were washed with phosphate-buffered saline (PBS) and incubated in a culture medium without serum for 48 h to allow exosome release. Subsequently, the supernatants were collected by centrifugation at 300 × *g* for 10 min, followed by that at 3000 × *g* for 20 min at 4 °C to remove cells and apoptotic bodies, followed by filtration through a 0.22 μm filter to remove cell debris. Next, the filtered supernatant was transferred to Amicon Ultra-15 Centrifugal Filter Units, MWCO 100 kDa (Millipore, Massachusetts, USA), and ultrafiltration liquid collected by centrifugation at 4000 × *g* for 30 min, mixed with exosome precipitation solution (ExoQuick-TC, System Biosciences, California, USA), and incubated at 4 °C overnight. After incubation, the final pellet was obtained by centrifugation of the samples at 1500 × *g* for 30 min at 4 °C. The final pellet containing exosomes was re-suspended in PBS for subsequent assays. The total exosomal protein concentrations were detected using the BCA Protein Assay kit (Thermo Fisher Scientific, Waltham, USA).

The size distribution and concentration of exosomes were measured by nanoparticle tracking analysis (NTA) using a Zeta View nanoparticle tracking analyzer (Particle Metrix, North Carolina, USA), and the exosome morphology was observed under a JEM-1400 transmission electron microscope (TEM) (Hitachi, Tokyo, Japan). The characteristics of exosome surface marker proteins Alix, TSG101, CD63, and CD9 were analyzed by Western blots.

### Detection of exosome transfer with the Cre-loxp system in vitro

MHCC-97H were stably transfected with GOLM1-Cre and THP-1 monocytes were stably transfected with pLV-CMV-LoxP-DsRed-LoxP-eGFP. THP-1 monocytes were differentiated into macrophages by incubation with 100 nM of phorbol 12-myristate-13-acetate (PMA) for 48 h. 200 μg Exosomes isolated from 5 × 10^7^ MHCC-97H-GOLM1-Cre cells were added to the medium of 1 × 10^7^ macrophages-LoxP-DsRed-LoxP-eGFP. The medium was not changed for 3 days to allow exosomes carrying GOLM1-Cre to be taken up by macrophages. The use of Cre by these macrophages would cause the excision of dsRed-stop (red) between the two loxp sites and the expression of GFP (green).

### Statistical analysis

All numerical values were represented as mean ± SEM. Depending on the data, Student’s t-test or one-way ANOVA was performed with GraphPad Prism 7.0 (GraphPad Software, Inc., San Diego, CA), with *p* < 0.05 defined as significant. Number of asterisks denote minimum statistical significance, i.e. *:*p* < 0.05, **:*p* < 0.01, and ***:*p* < 0.001, ns no significant. For more details and other methods, see supplementary information. Antibodies and reagents applied in this study are listed in Supplementary Table 1.

## Supplementary information

Supplementary Materials

## Data Availability

All data supporting the findings of this study are available from the corresponding author on reasonable request.
