# Supplementary material for: GOLM1 exacerbates CD8+ T cell suppression in hepatocellular carcinoma by promoting exosomal PD-L1 transport into tumor-associated macrophages
Source: Signal Transduct Target Ther. 2021 Nov 19;6:397. doi: 10.1038/s41392-021-00784-0 (PMC8602261; doi:10.1038/s41392-021-00784-0)
Supplement: Supplementary file 1 — Supplementary Materials [file 41392_2021_784_MOESM1_ESM.pdf]

Supplementary Materials for

GOLM1 Exacerbates CD8$^+$ T cell Suppression in Hepatocellular Carcinoma by Promoting Exosomal PD-L1 Transport into Tumor-associated Macrophages

Jinhong Chen,[1,3] Zhifei Lin,[1,3] Lu Liu[2,3], Rui Zhang,[1] Yan Geng,[1] Minghao Fan,[1] Wenwei Zhu,[1] Ming Lu,[1] Lu Lu,[1] Huliang Jia,[1] Jubo Zhang,[2,*] Lun-Xiu Qin[1,4,*]

Correspondence to: qinlx@fudan.edu.cn

This PDF file includes:

Materials and Methods
Figures. S1 to S6
Tables S1

Materials and Methods

Western blotting and immunoprecipitation

Total protein was extracted from cells, exosomes or tissues using RIPA buffer (Thermo Fisher Scientific, USA) containing protease inhibitor (Thermo Fisher Scientific, USA). The total protein concentrations were detected by using the BCA Protein Assay kit (Thermo Fisher Scientific, USA). Protein samples were separated by sodium dodecyl sulfate polyacrylamide gel electrophoresis (SDS-PAGE) and transferred to nitrocellulose filter membranes. After blocking with 5% fat-free milk in TBS-T at RT for 1h, membranes were incubated with the primary antibody at 4℃ overnight. Then, membranes were incubated with Peroxidase-conjugated secondary antibody (CST), and the antigen – antibody reaction was visualized by enhanced chemiluminescence assay (ECL, Thermo Fisher Scientific, USA). Image acquisition were performed using the Amersham Imager 600 RGB (General Electric Company, USA).

Immunoprecipitation was performed as described previously. In brief, whole cell lysates from HCC cell lines were incubated with anti-FLAG Magnetic beads (Sigma Aldrich) or antibody against PD-L1 and protein A/G Magnetic beads (Bimake) overnight, after six times wash with RIPA buffer, the proteins coupled to the gel were eluted or boiled in protein loading buffer, followed by SDS-PAGE separation and immunoblot with specific antibodies.

Immunohistochemistry

Immunohistochemistry was performed as described previously. Briefly, sections were deparaffinized in xylene (100%, three times, 5 min each) and rehydrated by stepwise washes in decreasing ethanol solutions ratio (100, 95, 80 and 75% for 5 min each) at room temperature. Slides were treated with 3% hydrogen peroxide for 15 min at room temperature. Antigen retrieval was achieved by boiling slides in the autoclave (80 kpa and 100˚C) for 1 min in 0.01 M sodium citrate buffer (pH 6.0), followed by cooling to room temperature. Sections were incubated with 10% normal goat serum (Boster Biological Technology, Pleasanton, CA, USA) for 60 min at room temperature to block nonspecific antigen sites. Slides were incubated with primary antibodies overnight at 4˚C, followed by incubation with the components of the Envision-plus detection system (EnVision +/HRP/Mo, Dako) for 60 min at room temperature. The sections were stained with 3,3'-diaminobenidine (DAB) for 1 min at room temperature and counterstained with hematoxylin for 1 min at room temperature. Negative controls were treated identically except without adding the primary antibodies. Immunohistochemistry staining was assessed by two independent pathologists with no prior knowledge of patient characteristics. Discrepancies were resolved by consensus.

The final staining score was ranked in four grades according to the values of immunoreaction intensity and percentage of cells staining: Score 0 (weak, less than the 25th percentile value), score 1 (moderate, between 25th and 50th percentile value), score 2 (strong, between 50th and 75th percentile value), and score 3 (very strong, higher than 75th percentile value). HCC tissues with staining scores 0 and 1 were defined as low expression, and those with scores 2 and 3 were defined as high expression (representative figures are demonstrated in Supplementary Figure 1).

Immunofluorescence

For immunofluorescence staining of cultured HCC cells, cells were seeded on coverslips and allowed to attach overnight. Adherent cells were fixed in 4% paraformaldehyde at room temperature for 20 min, permeabilized in 0.1% Triton X-100 for 10 min at 4℃, and then blocked

with 5% bovine serum albumin in PBS at room temperature for 45min. Cells were stained with primary antibodies overnight at 4 °C, followed by goat anti-mouse Alexa Fluor 594 dye conjugate (Abcam) and goat anti-rabbit Alexa Fluor 488 dye conjugate (Abcam) secondary antibodies incubation at room temperature for 1 h. Nuclei were stained with 4,6-diamidino-2-phenylindole (DAPI; Life Technologies). Images were captured using a confocal fluorescent microscope (Lecia Microsystems CMS GmbH Am Friedensplatz 3, Mannheim, Germany).

For immunofluorescence staining of tumor tissues from HCC patients or C57/BL mice, tumor masses were frozen in an optimal cutting temperature block immediately after extraction. Cryostat sections of samples that were 5 μm thick were attached to saline-coated slides. Cryostat sections were fixed with 4% paraformaldehyde for 20 min at room temperature and blocked with 5% bovine serum albumin in PBS at room temperature for 45 min. Sample sections were stained with primary antibodies overnight at 4 °C, followed by goat anti-mouse Alexa Fluor 594 dye conjugate (Abcam) and goat anti-rabbit Alexa Fluor 488 dye conjugate (Abcam) secondary antibodies incubation at room temperature for 1 hour. Nuclear staining was performed with DAPI. A confocal microscope (Lecia Microsystems CMS GmbH Am Friedensplatz 3, Mannheim, Germany) was used for image analysis.

Expression and purification of GST protein

Transform control construct and tag-fusion-constructs into E.coli BL21 (DE3). Recombinant E. coli clones were grown in LB medium (with 100 μg/ml ampicillin) at 37°C. Bacteria were induced for the expression of the genes cloned in pGEX4T1 with 1 mM IPTG (Genie, Bangalore) at an OD600 of 0.6. After an induction period of 4 h at 22 °C, the bacterial pellet was harvested, washed and resuspended in Potassium phosphate buffer (pH 7.2) supplemented with 0.2 M NaCl, 5 mM DTT and 2 mM EDTA. The bacterial pellet was lysed by sonication and the insoluble fraction along with cell debris was removed by centrifugation at 20,000g for 30 min. The supernatant was further subjected to affinity chromatography with the Glutathione Sepharose 4B (GE, USA) according to the manufacturer's instructions for protein purification. Fractions showing the GST-GOLM1 protein were pooled from glutathione Sepharose affinity chromatography step and further purified using pre-packed and calibrated SephacrylS400 16/60 size exclusion column (GE, USA). Analyses of expression and purification were performed by SDS-PAGE. Concentration of the purified protein was determined based on BCA Protein Assay kit (Thermo Fisher Scientific, USA).

GST Pull-down Assay

GST fusion proteins were immobilized on glutathione agarose beads; blocked with PBS containing 0.1% NP-40 (v/v), 5 mM EDTA, 5 mM EGTA, 20 mM leupeptin, 25 mg/ml ALLN, 5 mg/ml pepstatin A, and 2 mg/ml aprotinin plus 5% nonfat milk; and incubated with the indicated proteins for 2 hour at 4℃. After washing five times, the bound proteins were dissolved in SDS sample buffer, separated by SDS-PAGE, and subjected to immunoblot assays.

RNA Isolation and Quantitative reverse transcription (qRT) PCR assays

Total RNA was isolated from cells using Trizol reagent (Invitrogen) according to the manufacturer's instructions. The concentration of the isolated RNA and the ratio of absorbance at 260 nm to 280 nm (A260/A280 ratio) were measured with NanoDrop 2000c Spectrophotometer (Thermo Fisher Scientific, USA). The cDNA was synthesized using SuperScript™ First-Strand Synthesis System (Invitrogen) according to the manufacturer's

instructions (Invitrogen). All cDNA was stored at -20°C until RT-PCR analysis. For the RT PCR reaction, Quantitative RT-PCR was conducted on an ABI QuantStudio 6 flex Real-Time PCR System (Applied Biosystems, USA) using SYBR green (Thermo Fisher Scientific, USA). All data analysis was performed using the comparative Ct method. Results were first normalized to internal control β-actin mRNA.

Exosome co-culture with macrophages in vitro

THP-1 monocytes were differentiated into macrophages by incubation with 100 nM of phorbol 12-myristate-13-acetate (PMA) for 48 hours. Macrophages (2×106/well) were plated in 6-well plates and co-cultured with 100 μg/mL of exosomes. After co-incubation for 72 hours, cells were collected for western blot and immunofluorescence.

Cell Lines and cell culture

Five human HCC cell lines (HCC-LM3, MHCC-97H, Huh7, PLC, and Hep3B) and THP-1 monocytes were used in this study. MHCC-97H and HCC-LM3 were established from the same parent human HCC cell line MHCC97. They have a genetically identical background and stepwise increasing metastatic potentials. PLC and Hep3B cells were obtained from American Type Culture Collection (Rockville, USA). Huh7 cells, Hepa1-6 and THP-1 monocytes were obtained from Shanghai Cell Bank of Chinese Academy of Science (Shanghai, China). HCC cells were cultured in DMEM (GIBCO) supplemented with 10% fetal bovine serum (FBS, GIBCO). THP-1 monocytes were cultured in RPMI supplemented with 10% FBS. THP-1 monocytes were differentiated into macrophages by incubation with 100 nM of phorbol 12-myristate-13-acetate (PMA) for 48 hours.

Macrophages chemotactic migration assays

The migratory ability of macrophages was determined using 24-well migration assay inserts (Corning, NY, USA). The lower chamber was filled with the supernatants from HCC cells as a chemoattractant. Macrophages were seeded at 5×104 in serum-free medium in the upper chamber and incubated for 48h to migrate into the lower chamber. Macrophages in the upper chamber were carefully removed using cotton buds and macrophages that migrated to the underside of the membrane were fixed and stained with Giemsa (Sigma, St Louis, USA), imaged, and counted with a microscope (Leica, Wetzlar, Germany). All experiments were performed in duplicate, and the cell numbers from 5 representative photos of each group were used for statistical analysis.

Ubiquitination assays

HEK293T cells were transfected with hemagglutinin-tagged (HA) ubiquitin, Flag-PD-L1 with or without GOLM1. MHCC-97H cells with shNT or shGOLM1 were transfected with hemagglutinin-tagged (HA) ubiquitin. Ubiquitinated PD-L1 was immunoprecipitated and subjected to western blot analysis with antibody against ubiquitin or HA. Cells were treated with MG132 prior to ubiquitination analysis.

DNA Constructs and transfection

All shRNA lentiviral constructs listed in the paper were purchased from Sigma-Aldrich.

CSN5 lentiviral overexpression vector was obtained by cloning human CSN5 coding sequence into pCDH vector. Flag-PD-L1 lentiviral vector was obtained by cloning PD-L1 into c-

terminal FLAG tagged pCDH. To obtain GOLM1-Cre fusion protein, Cre was cloned into GOLM1-pCDH vector to generated GOLM1 fused Cre. pLV-CMV-LoxP-DsRed-LoxP-eGFP was purchased from Addgene (Addgene Plasmid #65726). Constructs encoding various FLAG-tagged truncated GOLM1 were constructed as described previously.

For generation of stable HCC cells and macrophages using lentiviral infection, lentiviruses were produced by co-transfecting HEK293T cells with a lentiviral expression plasmid combined with psPAX2 and pMD2.G vector using Fugene (Promega) according to the manufacturer's instructions. After 48h the culture supernatants containing viruses were harvested, filtered through 0.45 mm filters and used to infect HCC cells and macrophages. The stable cell lines were obtained by 3 μg/ml puromycin selection for 1 week.

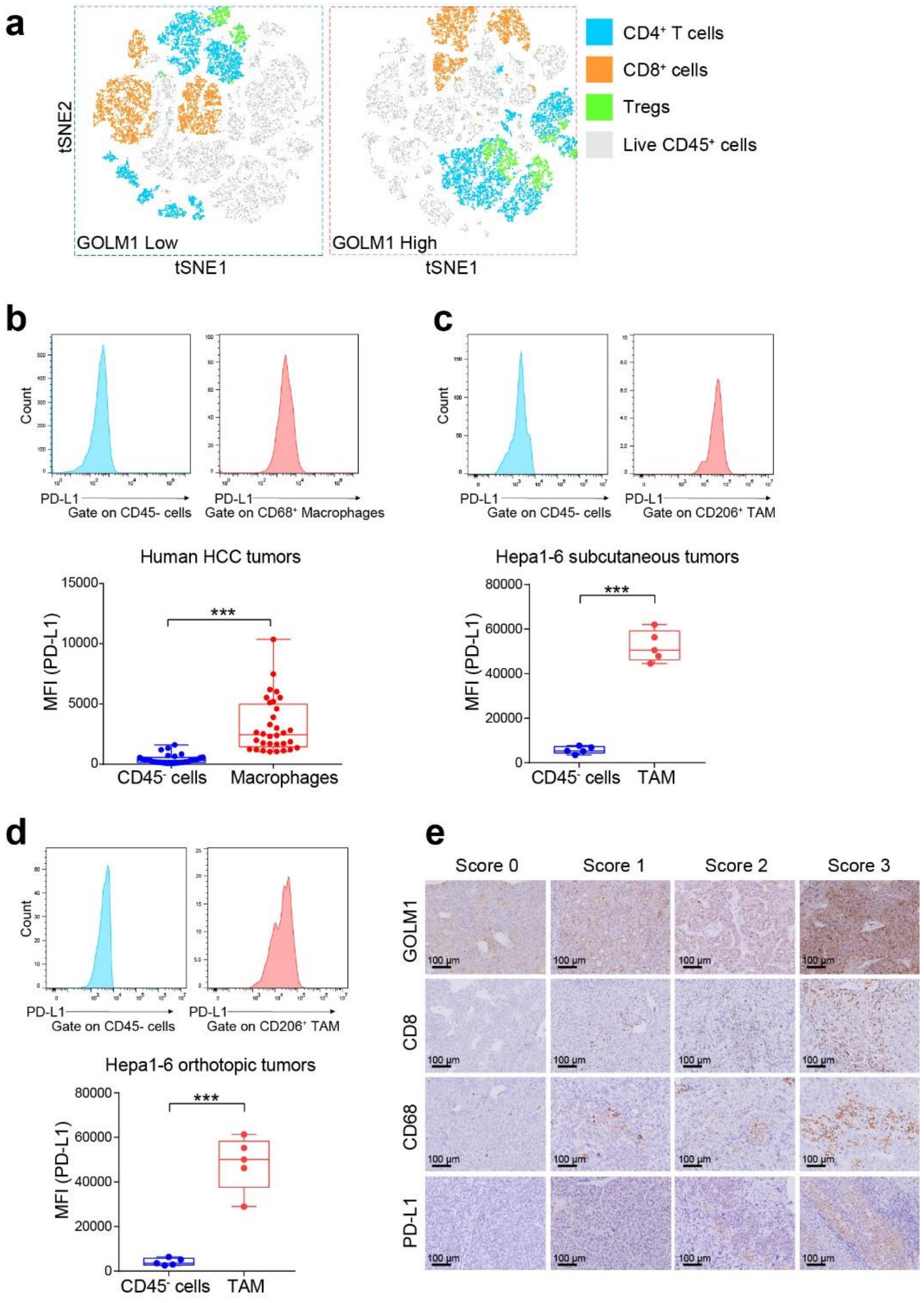

Figure. S1. PD-L1 levels on Macrophages are much higher than tumor cells in HCC.

(**a**) Representative tSNE map derived from flow cytometric analysis indicated the populations of the infiltrating immune cells, including $CD4^+$ T cells ($CD45^+CD3^+CD4^+$), $CD8^+$ T cells ($CD45^+CD3^+$ $CD8^+$), and Tregs ($CD45^+CD3^+CD4^+CD25^+FoxP3^+$).

(**b**) MFI of PD-L1 staining on tumor cells ($CD45^-$) and macrophages in human HCC tumors are shown (n = 32).

(**c-d**) MFI of PD-L1 staining on tumor cells ($CD45^-$) and TAM ($F4/80^+CD11b^+CD206^+$) in Hepa1-6 subcutaneous tumors (**c**), and Hepa1-6 orthotopic tumors (**d**) are shown (n = 5 per group).

(**e**) Scores indicate levels for GOLM1, PD-L1, CD8, and CD68 expression in representative human HCC tissues with IHC staining.

*** P<0.001.

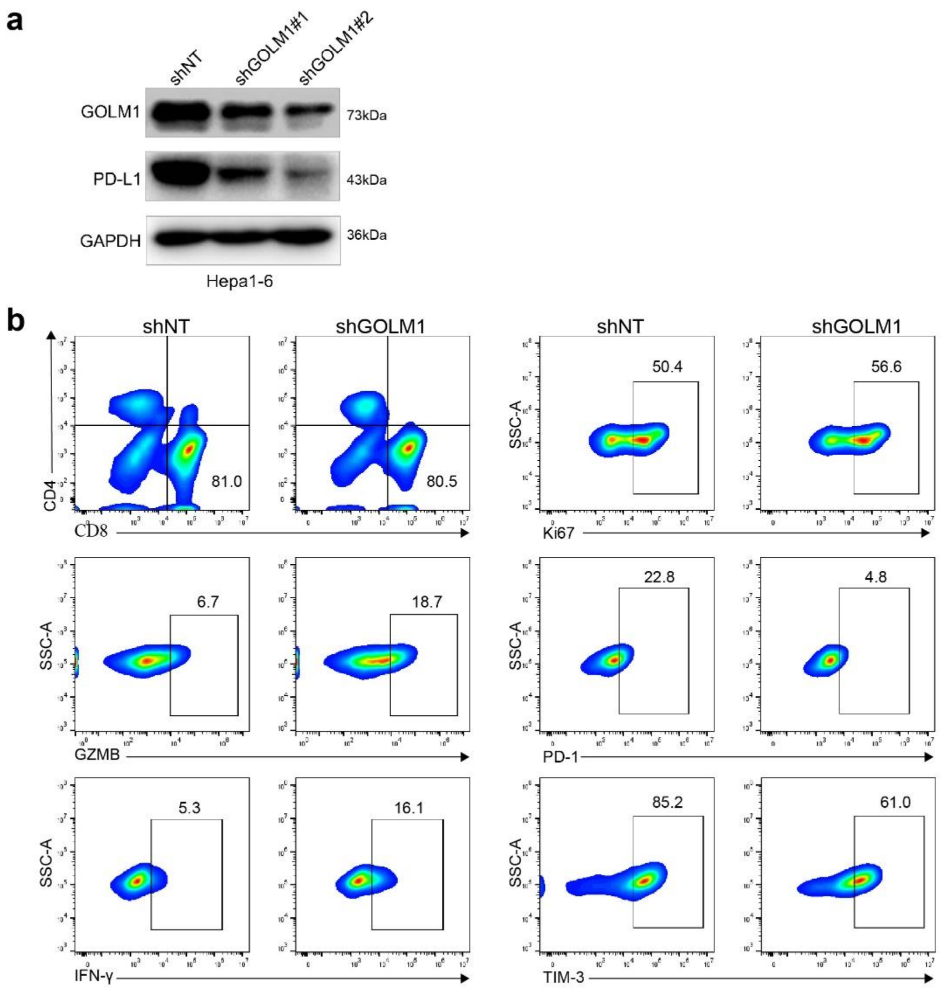

Figure. S2. GOLM1 upregulates PD-L1 expression and induces T-cell suppression in subcutaneous tumors.

(**a**) Western blotting demonstrated knockdown of GOLM1 decreased PD-L1 expression in mouse liver cancer cell line Hepa1-6 cells. The most significant knockdown effects on GOLM1 is induced by shGOLM1#2, which was adopted for further in vivo study in subcutaneous implantation model.

(**b**) Representative flow cytometry images of tumor-infiltrating CD8^{+} T cells and their functional makers (PD-1, TIM-3, IFN-y, GZMB and Ki67) in tumor tissues of subcutaneous implantation models of Hepa1-6 shNT and Hepa1-6-shGOLM1 cells.

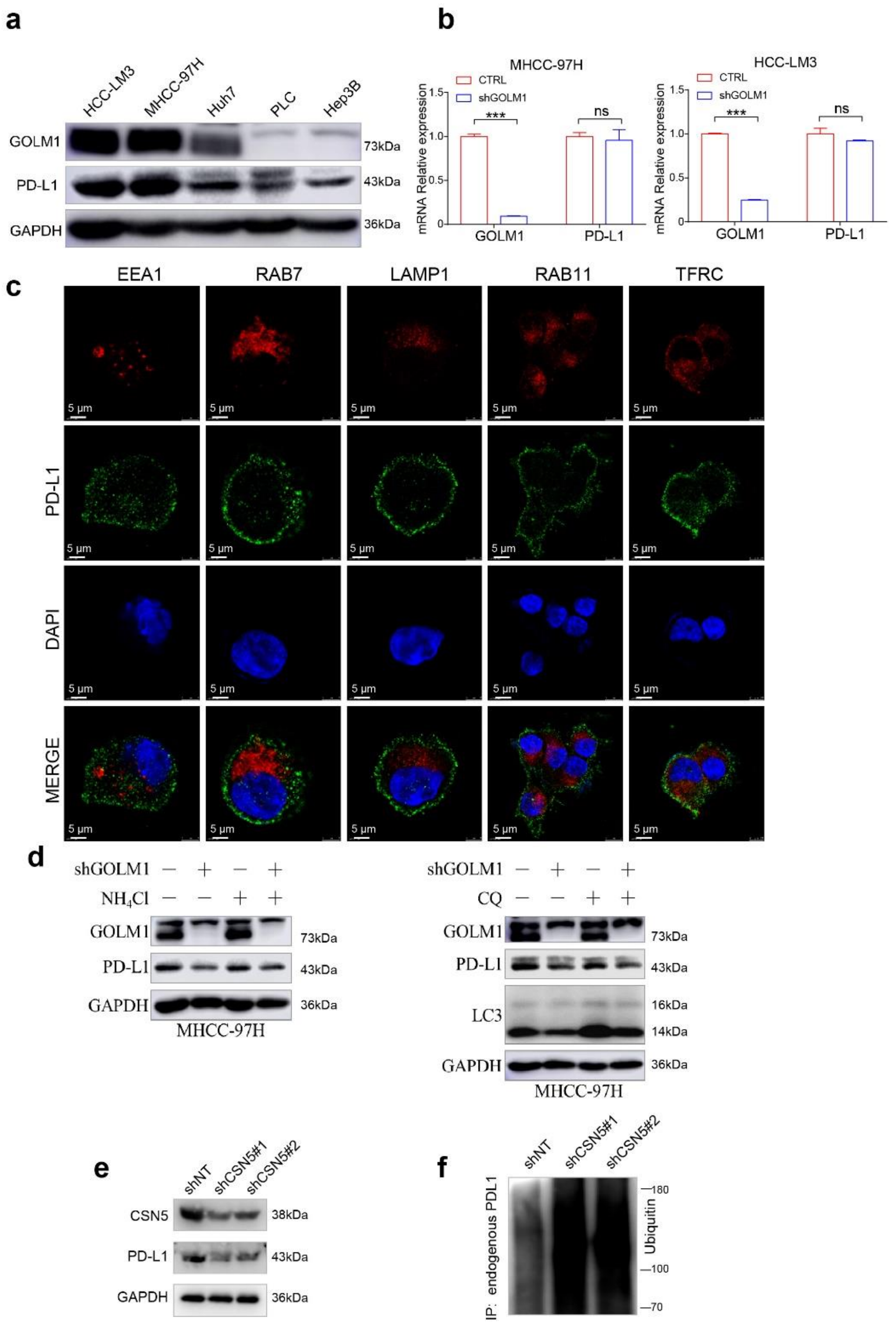

Figure. S3. Regulation of GOLM1 on PD-L1 expression was not at the transcriptional level or in the process of endocytosis and recycling.

(**a**) Western blotting analysis of GOLM1 and PD-L1 expression in different HCC cell lines. Significantly higher GOLM1 and PD-L1 levels were found consistently in HCC cell lines with higher metastatic capabilities (HCC-LM3 and MHCC-97H) compared with the others.

(**b**) GOLM1 knockdown had no effects on PD-L1 mRNA expression in MHCC-97H and HCC-LM3 cells quantified by qPCR.

(**c**) Immunofluorescence showed the subcellular compartments and markers co-stained with PD-L1. There was rare co-localization between PD-L1 and EEA1, Rab7, Rab11, Lamp1 and TFRC, respectively.

(**d**) Western blotting analysis showed the effect of GOLM1 on PD-L1 was not affected by the lysosomal inhibitors NH_4Cl (250μM) and chloroquine (CQ, 20μM). The level of LC3 was detected to show the activity of CQ.

(**e-f**) Western blotting analysis showed that CSN5-KD induced by shCSN5 resulted in a significant decrease of PD-L1 expression and increase of PD-L1 ubiquitination in PLC cells.

*** $P<0.001$, ns: no significant.

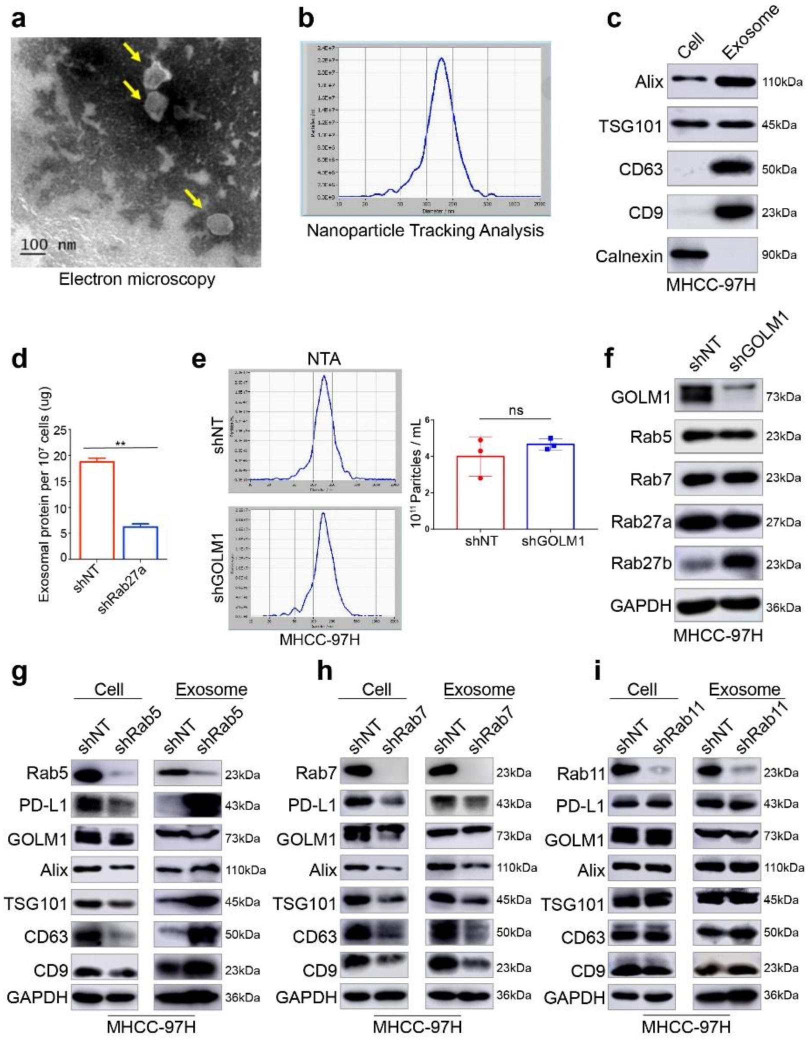

Figure. S4. Identification of exosomes and the effect of Rab family on exosomal PD-L1 secretion.

(**a**) The exosome morphologies were observed with transmission electron microscope (TEM).

(**b**) The size distribution and concentration of exosomes were measured by nanoparticle tracking analysis (NTA).

(**c**) The characteristics of exosome surface marker proteins Alix, CD9, CD63, and TSG101 were analyzed by Western blot.

(**d**) The total amounts of proteins in the exosomes purified from MHCC-97H cells with RAB27a knockdown were quantified by BCA assay. Data showed the amounts of exosomal proteins per 10 million secreting cells.

(**e**) The NTA showed no significant difference of exosome amount between shNT and shGOLM1 in MHCC-97H cells.

(**f**)Western blotting analysis showed GOLM1 has no effect on Rab5, Rab7, Rab27a, except for Rab27b.

(**g-i**) Western blotting analysis showed the effect of knockdown of Rab5, Rab7 and Rab11 on exosomal PD-L1 secretion in MHCC-97H cells. Knockdown of Rab5 reduced PD-L1 expression on the cell surface, but resulted in increased PD-L1 levels in exosomes with increased exosome amount (**g**). Knockdown of Rab7 simultaneously decreased PD-L1 and exosomal marker proteins in cells and exosomes (**h**). Knockdown of Rab11 had no effect on PD-L1 and exosome secretion (**i**).

** P<0.01, ns: no significant.

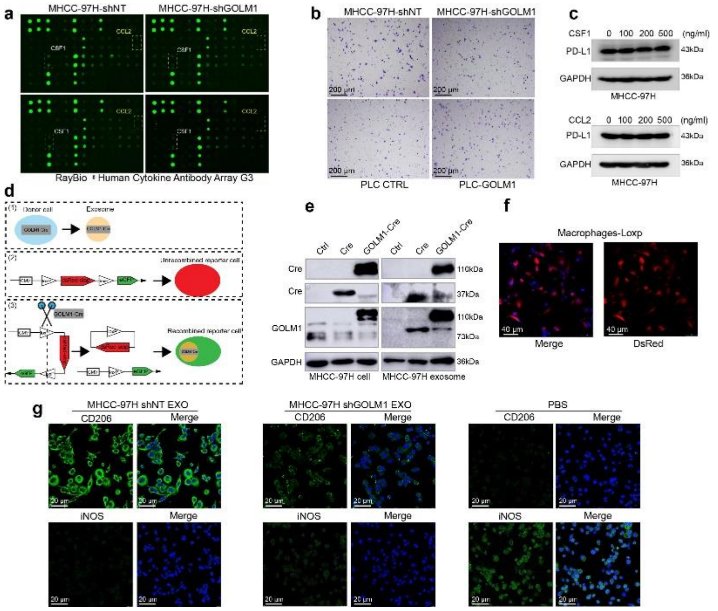

Figure. S5. HCC cells with High GOLM1 expression recruit and educate macrophages through CSF1 and exosomes.

(**a**) An antibody arrays of cytokines relating to recruit TAM showed CSF1 and CCL2 decreased significantly in the supernatant from MHCC-97H-shGOLM1 cells.

(**b**) Migration assays showed macrophages migration towards the supernatants of MHCC-97H-shGOLM1 or PLC-CTRL cells decreased significantly.

(**c**) Western blotting analysis showed that macrophages cultured with cytokines CSF1 and CCL2 didn’t increase the PD-L1 level in vitro.

(**d**) Schematic diagram showed the Cre-Loxp system as a method of exosomes transfer.

(**e**) Western blot of exosomes revealed successful transfection of GOLM1-Cre into MHCC-97H cells. The GOLM1-Cre fusion protein was 110 kDa.

(**f**) Immunofluorescence showed the successful transfection of LoxP-DsRed-Stop-LoxP-eGFP into macrophages, which can be detected in the red but not green channels.

(**g**) Immunofluorescence showed increased M2 marker CD206 and decreased M1 marker iNOS on macrophages cultured with MHCC-97H-shNT derived exosomes.

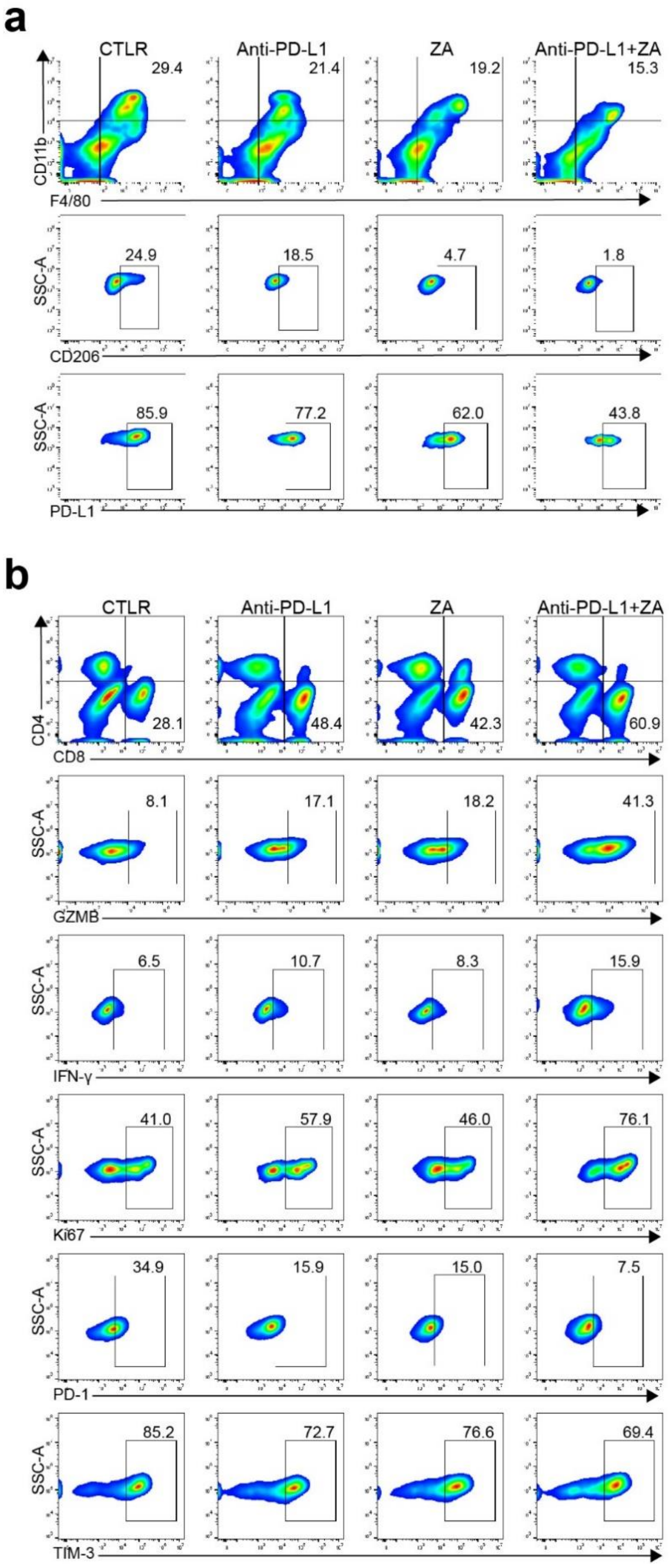
a
CTLR
Anti-PD-L1
ZA
Anti-PD-L1+ZA
29.4
21.4
19.2
15.3
CD11b
F4/80
24.9
18.5
4.7
1.8
SSC-A
CD206
85.9
77.2
62.0
43.8
PD-L1
b
28.1
48.4
42.3
60.9
CD4
CD8
8.1
17.1
18.2
41.3
GZMB
6.5
10.7
8.3
15.9
IFN-γ
41.0
57.9
46.0
76.1
Ki67
34.9
15.9
15.0
7.5
PD-1
85.2
72.7
76.6
69.4
TIM-3

Figure. S6. ZA and anti-PD-L1 combination decreases PD-L1$^+$ TAM infiltration and ameliorates T cell suppression in HCC.

(**a**) Representative flow cytometry images of the infiltrated macrophages (F4/80$^+$ CD11b$^+$), TAMs (F4/80$^+$ CD11b$^+$ CD206$^+$) and PD-L1$^+$ TAMs (F4/80$^+$ CD11b$^+$ CD206$^+$ PD-L1$^+$) in orthotopic tumors from each group.

(**b**) Representative flow cytometry images of tumor-infiltrating CD8$^+$ T cells and their functional makers (GZMB, IFN-y, Ki67, PD-1 and TIM-3) in orthotopic tumors from each group.

Table S1. Reagent or resource

REAGENT or RESOURCE	SOURCE	IDENTIFIER
Antibodies		
Rabbit monoclonal Anti-GOLM1	Abcam	Cat#ab109628，RRID:AB_10860116
Rabbit monoclonal Anti-PD-L1	Cell Signaling Technology	Cat#13684, RRID:AB_2687655
Mouse monoclonal Anti-GAPDH	Cell Signaling Technology	Cat#5174S, RRID:AB_10622025
Mouse monoclonal Anti-Actin	Proteintech	Cat#60008-1-Ig, RRID:AB_2289225
Mouse monoclonal Anti-CD68	Abcam	Cat#ab955, RRID:AB_307338
Rabbit monoclonal Anti-CD8	Abcam	Cat#ab101500
Mouse monoclonal anti-PD-L1	Proteintech	Cat#66248-1-Ig
Rabbit monoclonal Anti-PD-L1	Abcam	Cat#ab205921, RRID:AB_2687878
Rabbit monoclonal Anti-F4/80	Cell Signaling Technology	Cat#70076S, RRID:AB_2799771
Rabbit polyclonal Anti-CD206	Abcam	Cat#ab64693
Rabbit polyclonal Anti-Flag	Sigma-Aldrich	Cat#F7425, RRID:AB_439687
Rabbit monoclonal Anti-HA	Cell Signaling Technology	Cat#3724
Mouse monoclonal Anti-Ubiquitin	Cell Signaling Technology	Cat#3936, RRID:AB_331292
Rabbit monoclonal Anti-CSN5	Abcam	Cat#ab124720, RRID:AB_10974089
Mouse monoclonal Anti-CD63	Biolegend	Cat#353013, RRID:AB_2561404
Rabbit polyclonal Anti-CD63	Proteintech	Cat#25682-1-AP, RRID:AB_2783831
Mouse Monoclonal Anti-Alix	Abcam	Cat#ab117600
Mouse Monoclonal Anti-CD9	Proteintech	Cat#60232-1-Ig, RRID:AB_11232215
Rabbit Polyclonal Anti-TSG101	Proteintech	Cat#14497-1-AP, RRID:AB_2208090
Rabbit monoclonal Anti-Calnexin	Abcam	Cat#ab133615
Mouse Monoclonal Anti-Rab27a	Proteintech	Cat#66058-1-Ig, RRID:AB_11042596
Rabbit Polyclonal Anti-Rab27b	Proteintech	Cat#13412-1-AP, RRID:AB_2176732
Rabbit Polyclonal Anti-CSF1	Proteintech	Cat#14779-1-AP, RRID:AB_10643241
Mouse monoclonal Anti-CCL2	Proteintech	Cat#66272-1-Ig, RRID:AB_2861337
Rabbit monoclonal Anti-Cre	Cell Signaling Technology	Cat#15036, RRID:AB_2798694
Mouse Monoclonal Anti-TGN46	Abcam	Cat#ab2809, RRID:AB_2203290

Mouse Monoclonal Anti-EEA1	BD Biosciences	Cat#610456, RRID:AB_397829
Mouse Monoclonal Anti-Rab5	Cell Signaling Technology	Cat#46449, RRID:AB_2799303
Mouse Monoclonal Anti-Rab7	Santa Cruz Biotechnology	Cat#Sc-376362
Mouse Monoclonal Anti-Rab11	BD Biosciences	Cat#610656, RRID:AB_397983
Mouse Monoclonal Anti-LAMP1	Abcam	Cat#ab25630, RRID:AB_470708
Mouse Monoclonal Anti-TFRC	Life technologies	Cat#A11130
Rabbit monoclonal Anti-iNOS	Abcam	Cat#ab178945
Rabbit monoclonal Anti-LC3B	Cell Signaling Technology	Cat#3868, RRID:AB_2137707
APC anti-human CD274 Antibody for FC	Biolegend	Cat#329708
Goat polyclonal Secondary Antibody to Rabbit IgG - H&L (Alexa Fluor® 488)	Abcam	Cat#ab150077, RRID:AB_2630356
Goat polyclonal Secondary Antibody to Mouse IgG - H&L (Alexa Fluor® 594)	Abcam	Cat#ab150116, RRID:AB_2650601
CD45 Monoclonal Antibody (HI30), FITC	BD Pharmingen	Cat#555482, RRID:AB_10371768
Alexa Fluor® 700 Mouse Anti-Human CD14	BD Pharmingen	Cat#561029
BV421 Mouse Anti-Human CD68	BD Pharmingen	Cat#564943
BV510 Mouse Anti-Human CD16	BD Pharmingen	Cat#563829
BV650 Mouse Anti-Human CD56	BD Pharmingen	Cat#564057
BV605 Mouse Anti-Human CD19	BD Pharmingen	Cat#562654
E-Cy™7 Mouse Anti-Human CD11b	BD Pharmingen	Cat#561685
PE Mouse Anti-Human CD33	BD Pharmingen	Cat#561816
PerCP-Cy™5.5 Mouse Anti-Human CD3	BD Pharmingen	Cat#560835
Alexa Fluor® 700 Mouse Anti-Human CD4	BD Pharmingen	Cat#561030
BV605 Mouse Anti-Human CD8	BD Pharmingen	Cat#564115
PE-Cy™7 Mouse Anti-Human CD25	BD Pharmingen	Cat#560920
Alexa Fluor® 647 Mouse anti-Human FoxP3	BD Pharmingen	Cat#560889
PE Mouse Anti-Human CD279	BD Pharmingen	Cat#560908
BV421 Mouse Anti-Ki-67	BD Pharmingen	Cat#565929
BV510 Mouse Anti-Human CD45RA	BD Pharmingen	Cat#563031
FITC Rat Anti-Mouse CD45	BD Pharmingen	Cat#561088
BV510 Rat Anti-Mouse CD8a	BD Pharmingen	Cat#563068
APC-R700 Hamster Anti-Mouse CD279	BD Pharmingen	Cat#565815
PE Mouse Anti-Mouse CD366 (TIM-3)	BD Pharmingen	Cat#566346
BV650 Rat Anti-Mouse IFN-γ	BD Pharmingen	Cat#563854
PE-Cy™7 Mouse anti-Ki-67	BD Pharmingen	Cat#561283
Alexa Fluor 647 anti-human/mouse Granzyme B	Biolegend	Cat#515406
BB700 Rat Anti-Mouse CD4	BD Pharmingen	Cat#566408
BV421 Rat Anti-Mouse CD274	BD Pharmingen	Cat#564716
PE Rat Anti-Mouse F4/80	BD Pharmingen	Cat#565410
PE-Cy™7 Rat Anti-CD11b	BD Pharmingen	Cat#561098
BV786 Hamster Anti-Mouse CD11c	BD Pharmingen	Cat#563735
Alexa Fluor® 647 Rat Anti-Mouse CD206	BD Pharmingen	Cat#565250

IFN gamma Monoclonal Antibody (B27), Alexa Fluor 700	Biolegend	Cat#557995, RRID:AB_2539767
BV650 Mouse Anti-Human TIM-3 (CD366)	BD Pharmingen	Cat#565564
PE/Cy7 anti-human/mouse Granzyme B Recombinant	BD Pharmingen	Cat#72214
Alexa Fluor® 647 Anti-Active Caspase-3	BD Pharmingen	Cat#560626
Chemicals, Peptides, and Recombinant Proteins		
BCA protein assay	Pierce	23225
Protease inhibitor	Bimake	B14001
Immobilon western chemilum HRP substrate	Merck Millipore	WBKLS0500
FuGENE™ HD Transfection Reagent	Promega	Cat#E2311
Protein A/G PLUS-agarose	Santa cruz	sc-2003
Puromycin, dihydrochloride	CAISSON	P067
Chloroform	Amresco	0757
Methanol	Sigma-Aldrich	14262
MG132	Selleck	S2619
Anti-FLAG® M2 Magnetic Beads antibody	Sigma-Aldrich	Cat# M8823, RRID:AB_2637089
Cycloheximide	R&D Systems	Cat#0970
Critical Commercial Assays		
ExoQuick-TC	System Biosciences	Cat#EXoTC10A-1
Experimental Models: Cell Lines		
Human: MHCC-97H	Established by Liver Cancer Institute, Fudan University (Shanghai, China)	N/A
Human: HCC-LM3	Established by Liver Cancer Institute, Fudan University (Shanghai, China)	N/A
Mouse: Hepa1-6	Shanghai Cell Bank of Chinese Academy of Science (Shanghai, China)	N/A
Human: THP-1	Shanghai Cell Bank of Chinese Academy of Science (Shanghai, China)	N/A
Human: Huh7	Shanghai Cell Bank of Chinese Academy of Science (Shanghai, China)	N/A
Human: PLC	ATCC	CRL-8024™
Human: Hep3B	ATCC	HB-8064™
